# Identifying barriers and potential solutions to improve equitable access to community eye services in central Kenya: a rapid exploratory sequential mixed methods study

**DOI:** 10.1136/bmjph-2025-002856

**Published:** 2026-09-21

**Authors:** Luke N Allen, Sarah Karanja, Michael Gichangi, Cosmas Bunywera, Emmaculate Muturi, Dickson Gachobi, Purity Kathure, Elizabeth Mutile Muasa, Lorna Mutwiri, Lorna Kajuju, Faith Kagwiria, Benjamin Ntabathia, Hillary Rono, David Macleod, Min Kim, Malebogo Tlhajoane, Matthew J Burton, Jacqueline Ramke, Nigel M Bolster, Andrew Bastawrous

**Affiliations:** 1 Nuffield Department of Primary Care Health Sciences, University of Oxford, Oxford, UK; 2 Department of Clinical Research, London School of Hygiene & Tropical Medicine, London, UK; 3 Centre for Public Health Research, Kenya Medical Research Institute, Nairobi, Kenya; 4 Ophthalmic Services Unit, Kenya Ministry of Health, Nairobi, Kenya; 5 Peek Vision Ltd, Kitale, Kenya; 6 College of Ophthalmology of Eastern Central and Southern Africa, Nairobi, Kenya; 7 Lifecare Hospital, Nairobi, Kenya; 8 Meru County Health Department, Meru, Kenya; 9 Chuka University, Chuka, Eastern, Kenya; 10 Kitale County District Hospital, Kitale, Kenya; 11 Department of Infectious Disease Epidemiology and International Health, London School of Hygiene & Tropical Medicine, London, UK; 12 University of Auckland, Auckland, New Zealand; 13 Peek Vision Ltd, London, UK

**Keywords:** Public Health, Community Health, Health Services Accessibility, Qualitative Research

## Abstract

**Background:**

Recent research has found that less than half of people identified with an eye problem in Meru county’s screening programme were able to access care, with younger adults being the least likely to receive the care they needed. We aimed to interview and survey members of this ‘left-behind’ group to explore barriers and identify potential solutions using a rapid mixed-methods approach.

**Methods:**

First, we conducted interviews to explore perceptions of barriers and potential solutions. Next, we asked a representative sample to rank the suggested solutions by likely impact. Finally, we held a multistakeholder workshop to identify which of the top-ranked interventions offered the best balance of impact, feasibility, cost and potential risks. We used a deductive matrix and thematic analysis to rapidly analyse the interview data.

**Results:**

We conducted 67 interviews. Barriers to access included long queues, conflicting work engagements and lack of clear information. Proposed solutions focused on reducing queue lengths, providing better counselling and clinic information, holding mop-up clinics and maintaining adequate stocks and supplies. We conducted ranking surveys with 401 additional people from the left-behind group. All proposed solutions were ranked at moderately-to-highly likely to improve equitable access. Fifteen people attended the multistakeholder meeting, including community representatives. Workshop participants unanimously selected enhanced counselling and SMS reminders as the interventions that offered the best balance of impact, risk, cost and feasibility. The other proposed solutions were deemed impractical or unaffordable.

**Conclusions:**

Rapid mixed-methods and multistakeholder collaboration were used to identify a range of potential service modifications that will be implemented within the ongoing programme. Our approach was centred on the experiences and perceptions of those who face the highest barriers to care.

**WHAT IS ALREADY KNOWN ON THIS TOPIC**

- Across the African continent, approximately half of all ambulatory appointments are missed across all specialties and sociodemographic inequalities are ubiquitous. Work in Meru County, Kenya has shown that younger adults are less likely than any other sociodemographic group to access care at their local treatment clinic but it is not clear what the specific barriers are for this group.

**WHAT THIS STUDY ADDS**

- Having already identified younger adults as the least likely group to receive care in Meru County, this study introduces a novel mixed-methods approach for engaging with members of this left-behind group to rapidly identify barriers and scalable solutions. This is an approach that any community-based programme could use to generate robust, non-tokenistic insights from affected communities within a matter of weeks, minimising the research time requirement and number of senior researchers required whilst maintaining rigorous scientific standards.

**HOW THIS STUDY MIGHT AFFECT RESEARCH, PRACTICE OR POLICY**

- Equitably advancing universal health coverage is predicated on identifying and overcoming unique barriers to care; however, existing efforts rarely involve consultation or co-creation with affected communities. Building on existing rapid qualitative and mixed-methods methods, we have developed a cutting-edge approach to identify barriers, prioritise solutions and identify service modifications that are feasible to introduce. Our research group will use an embedded randomised controlled trial to implement and test suggested interventions, in the context of an equity-focused continuous improvement model.

## Introduction

 Improving equitable access to community health services lies at the heart of universal health coverage (UHC), and ‘leaving no one behind’ is the ‘central, transformative promise’ of the Sustainable Development Goals.^1–4^ WHO’s Thirteenth General Programme of Work states that ‘the main challenge to making progress towards UHC comes from persistent barriers to accessing health services’.^4^

Our research collaborative is developing and testing a new way to identify and address inequitable access to care using the ‘FAIR’ approach. This involves four distinctive steps:

1. Find cases and track referrals to identify all those with a health need.

2. Analyse attendance data to identify which groups are least likely to receive care.

3. Interview & survey people from these groups to identify and rank potential solutions.

4. Randomise the implementation of top-ranked solutions to test whether they equitably improve access to care.^5^ We are applying this model in the context of community-based eye screening programmes in Botswana, India, Kenya and Nepal.

Uncorrected visual impairment affects over a billion people worldwide, levying major social and economic costs, despite the availability of highly cost-effective treatments like spectacles and cataract surgery.^6^ Our first set of findings from a cross-sectional equity analysis of over 4000 people in Kenya’s Meru county—steps 1 and 2 in the FAIR approach—found that only 46.0% (95% CI 44.5% to 47.5%) of those found to have an eye care need were able to access their free local treatment outreach clinic.^7^ We found that younger adults, males and those working in sales, services or manual jobs were the least likely to receive the care they need. Age was the strongest predictor of poor access, with less than a third of people aged 18–44 years receiving care compared with two-thirds of those aged >45 years, even after controlling for severity of eye condition and a wide range of other factors.

Traditionally, ideas for how to improve programmes come from ‘experts’, service providers, or surveys of service users rather than affected people themselves.^8,9^ In the context of renewed interest in primary care^10–12^ and the insidious persistence of colonialism and epistemic injustice in global health,^13–15^ increasing attention is being paid to person-centredness and community-centredness. Simply put, advancing equitable access to health services must be done with, rather than to or on behalf of left-behind groups.^9^

In this study, we aimed to engage with younger adults who had not been able to access eye care in Meru in order to explore their perceptions of how the local services could be modified to improve access. Building on our equity analysis^7^ and working within the same live community screening programme, we aimed to deliver robust, non-tokenistic and generalisable findings within a matter of weeks, with a view to testing suggested service modifications with a subsequent embedded RCT.

## Methods

### Setting

Meru is a county with a population of 1.5 million people in central Kenya, 110 miles north of Nairobi. It includes Mount Kenya and Meru National Park. The capital, Meru town, is home to a quarter of a million people. Agriculture is the main source of employment, with khat and tea representing important cash crops. Eye services are available in urban centres, but are not free. Kenya’s Vision Impact Programme (‘VIP’) has been operating in Meru since July 2022, and has reached over 350 000 people to date, according to internal data. Teams of screeners go house-to-house testing all adults’ vision using a simple smartphone-based app developed by Peek Vision.^16^ Screeners refer people whose visual acuity falls below 6/12; those who have a red eye or another issue on basic visual inspection; and anyone who feels they have an eye problem, even if there are no clinical signs and their visual acuity is >6/12. Screening, referral and subsequent clinic assessment are all free. Our research team has been working with screeners to gather sociodemographic data from every person who screened positive and was referred to an outreach clinic for further assessment and treatment between April and July 2023. As stated above, we had previously found that younger adults are the least likely to be checked in at treatment clinics but we did not know what the main barriers were or what could be done.

### Research paradigm, theory and methodology

We used a pragmatist philosophical paradigm, which holds that methodological choices should be driven by the research question rather than by adherence to a single paradigm. Our research question required both qualitative depth (to generate barriers and solutions from lived experience) and quantitative breadth (to assess generalisability across the left-behind population), hence our use of a sequential mixed-methods design.^17,18^ We grounded our work in two complementary conceptual frameworks. Levesque *et al* conceptualise access as the interaction between five supply-side characteristics of services (approachability, acceptability, availability and accommodation, affordability and appropriateness) and five corresponding demand-side abilities of service users.^19^ Obrist *et al*’s Health Access Livelihood Framework shares these five dimensions but situates them explicitly within the context of livelihood insecurity, arguing that access is mediated by the capital assets that potential service users can mobilise.^20^ Together, these frameworks directed our attention to both programme-modifiable service characteristics and the structural determinants of users’ ability to engage with services. This distinction is helpful as our ultimate aim was to identify service modifications that improve accessibility for younger adults. We required mixed methods to answer a multilayered question: what are the main barriers to accessing eye services and what could be done about them?

### Methods overview

This study was conducted in three stages. In stage 1, we used interviews to generate a long-list of perceived barriers and potential solutions. In stage 2, to move from subjective experiences to generalisable service modifications, we conducted a telephone survey where a representative sample of younger adults who did not receive care ranked each of the suggested solutions by likely impact. Finally, in stage 3, these ranked solutions were reviewed by a multistakeholder group who identified a package of interventions to test based on likely impact, feasibility, cost and risks.

### Team composition and reflexivity

This project was part of the broader ‘IM-SEEN’ programme of work that seeks to develop a new, rapid, robust and responsive approach to continuously improving access to care, starting in the field of community-based eye screening programmes in Botswana, India, Kenya, Nepal. London School of Hygiene and Tropical Medicine (LSHTM)-based researchers (LNA, AB, MJB, JR, DM and MK) working with Kenya’s Ministry of Health eye lead MG, AB and NMB from Peek Vision—the screening programme software provider, and SK—the local research lead SK based at KEMRI, had already conducted a collaborative equity assessment of Meru’s VIP programme. LNA—a mid-career British clinician, policy advisor and mixed-methods public health researcher-led the development of the methodological approach to be used in all countries to engage with members of the left behind groups. LNA worked closely with SK—a mid-career female Kenyan public health social scientist—to tailor the approach for Meru County, supported by the wider team. LNA and SK recruited and trained six local, early-career data collectors (DG, EMM, EM, PK, BN and FK) to conduct the interviews and surveys. We were interested in understanding the barriers and solutions as perceived and described by affected people in their own words. SK and LNA facilitated the multistakeholder workshop where findings were interpreted by lay representatives, other members of the left behind group and local programme managers. This local multistakeholder group collectively made the final decisions on which suggested service modifications to take forward for implementation.

### Stage 1: interviews

#### Recruitment and sample size

Peek Vision—the programme software provider—provided us with a list of 517 people aged 18–44 years from the original equity analysis who had not been able to access their clinic appointment in Meru and who had provided consent to be contacted again. The equity analysis had found an attendance rate of 32.3% (95% CI 30.1% to 34.7%) among this age group.^7^ Every person screened in the programme had already provided a contact number. In random order, we phoned people from this list to invite them to participate in the interviews and sought recorded verbal informed consent. We tried to contact each person three times before moving on to the next person on the list.

We planned to use Guest *et al* approach to determine our sample size based on thematic saturation, using a ‘base’ of 12 interviews followed by runs of two interviews with a 0% new information threshold.^21^ In other words, we aimed to continue recruiting interviewees until no new themes emerged after two interviews in a row, with a minimum sample size of 14 (we dub this the ‘12+2’ approach).

#### Interview modality

We originally aimed to use telephone interviews, based on empirical evidence that they can be completed faster at lower cost than in-person interviews and with equivalent data richness.^22–25^ However, we were not entirely convinced that the data would be equivalent. As such, we decided to recruit two separate samples and use both modalities, conducting an embedded mode-comparison study^26^ that has been reported elsewhere.^27^

#### Data collection

Three pairs of Kenyan data collectors with at least basic qualitative training and fluency in English, Kiswahili and the local dialect were trained by LNA and SK to conduct semistructured interviews using the topic guide summarised in box 1 (see online supplemental appendix 1 for the full script). The topic guide was translated into Kiswahili and the local Meru dialect by the data collection team and back translated into English to ensure accuracy. For the telephone interviews, calls were made on speakerphone in a private space and recorded using the phone’s built-in call recording app. As one data collector conducted the interview, the other noted down the times at which each unique barrier and potential solution was mentioned. After the call, the interview recording was immediately replayed and the data collectors entered verbatim quotes directly from the audio into our analytic matrix. The same process was used for in-person interviews, but with an audio recorder instead of a mobile phone. Direct-from-audio transcription avoids the latency and potential loss of contextual detail associated with written transcription while maintaining verbatim fidelity to participants’ words.^28^ Interviewees did not review their transcribed quotes. In-person interviews were conducted in private rooms in four different health facilities where interviewees’ responses could not be overheard. Only the data collectors and the interviewee were present for each interview.

Box 1 **Topic guide**

**Barriers**

- In your own words, can you talk me through why we didn’t see you at that clinic?
  Probing questions:

- Are there any other factors that prevented you from attending?
- Is there anything else you’d like to share?

**Solutions**

- What would make the biggest difference in addressing these barriers?

  Probing questions:

- What else would help?
- What other changes could we make to the programme that would make it easier for you to attend?
- Are there any other specific changes that we could make to the way that the programme or eye clinics run?
  - You mentioned [list their proposed solutions]. Some of these may be beyond our control, but if we managed to [list their proposed programme-related changes], do you think that would be enough?
  - That’s the end of my questions. Is there anything else you would like to add

### Data analysis

We used an abductive analytic approach,^29^ whereby data collectors initially entered verbatim quotes relating to barriers and solutions into a deductive framework matrix, nesting each quote under one of ten broad a priori themes derived from the Levesque and Obrist access frameworks and a broader review of the literature, described fully in our protocol^30^:

- Costs.
- Distance and transport.
- Desire/priority to seek care.
- Clinical service quality.
- Facilities.
- Awareness and communication.
- Fear.
- Norms, values, health beliefs.
- Empowerment, support and capacity.
- Other (making room for surprising/unexpected themes).

At daily debrief sessions, SK and LNA reviewed the matrix with the data collectors and used inductive coding to identify unique barriers and solutions. The decision to use an analytic matrix and collective interpretation was based on our previous systematic review, which had found these techniques to be rapid and robust.^28^

Our matrix had one participant per column and one subtheme per row—with a new row created every time a sub-theme (a unique barrier or solution) was identified. Each sub-theme (eg, ‘loss of earnings’) was nested under the relevant theme (eg, ‘costs’). The process of data entry is demonstrated in this short online video (http://tinyurl.com/29asc6nm) and a blank matrix template is available here.

We generated one matrix for the telephone interviews and another for the in-person interviews. This was so that we could compare the themes that emerged from each modality in our embedded mode comparison study.^27^ For our main analysis, presented here, we pooled all barriers and solutions identified using both modalities.

We trained the data collectors over two days and performed fourteen pilot telephone interviews before starting data collection. Videocall debriefing sessions were held at the end of each day.

### Additional analysis

Our original equity analysis had also indicated that people with the highest incomes and those who owned a car or truck may have been less likely to attend than those reporting no vehicle ownership and lower incomes.^7^ We conducted an additional ten interviews with people who reported earnings in the highest income category to assess whether the barriers they reported differed from those reported by younger adults. We hypothesised that richer people did not access the service because they had sought private care after being identified with an eye need during eye screening.

### Output and screening

We created a summary list of all of the unique solutions that had emerged from the interviews. Before taking these to a representative sample for ranking, we met with the implementing partner to identify any ideas that would be completely infeasible given the constraints of the programme, for example, providing helicopter transportation. Any interventions that were deemed to be completely infeasible were removed from the list. We asked the director of Peek Vision to independently review each of these decisions.

### Stage 2: telephone survey

#### Survey instrument

We used the vetted list of solutions to generate a simple telephone-based survey (online supplemental appendix 2) where respondents were asked to rank each suggestion from 1 to 3 on a Likert scale:

1. It would make a small difference or no difference—that is, it might help a few people, but the impact is likely to be minimal,
2. It would make a moderate difference—that is, it would greatly increase the chances, but it would not be enough to guarantee attendance by itself,
3. It would make a big difference—that is, if we introduced this change then you or people like you would definitely attend.

The telephone ranking survey was piloted with 26 people.

#### Sampling and recruitment

We used a 95% CI, a 5% margin of error, and a conservative assumption that the total population size was 1 million people, rendering a minimum sample size of 384. We took the same list of 18–44 year-olds who had not been able to access care, and used random numbers to generate a call order, removing those who had already been included in the qualitative interviews. The same six data collectors tried calling each person three times before moving on to the next.

#### Data collection and analysis

Data collectors obtained recorded informed verbal consent, and then read through the survey instrument using an online data entry form. Data collectors entered the respondents’ scores for each proposed solution. We calculated the simple mean for each solution, and then ranked solutions by mean score.

#### Stage 3: multistakeholder workshop

**Figure 1:**
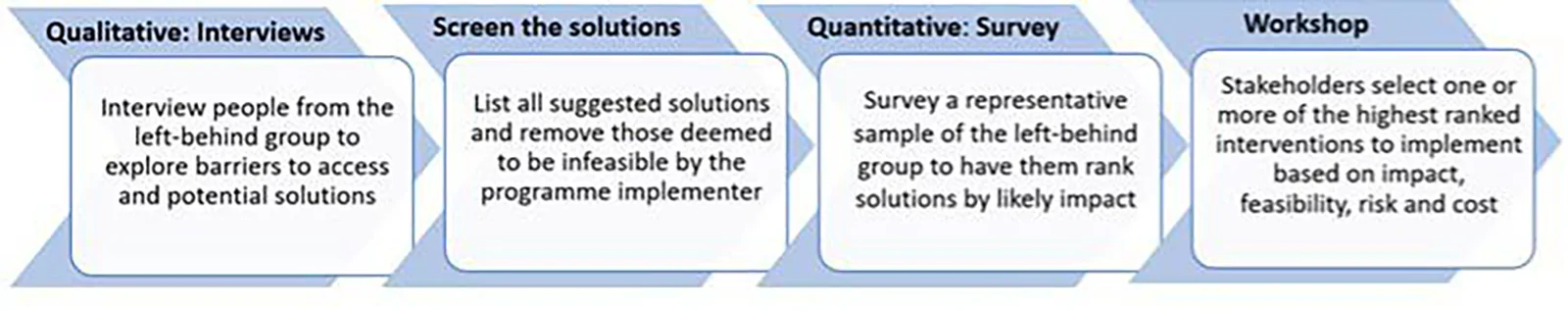
Accumulation of themes as the interviews progressed.

Once we had this ranked list of solutions, we convened an online workshop with representatives from the screening programme implementing partner organisation, the programme funder, the national eye screening programme office, Kenya Society for the Blind, the county health department, and members of our community advisory board (comprised of local patients and community leaders). We facilitated a discussion where each stakeholder shared their perceptions of the likely impact, feasibility, costs and risks associated with each solution. LNA and SK restricted their contributions to presenting the ranked solution scores, facilitating the discussion, and providing information on the general strength of the international research evidence for each of the proposed solutions. At the end of the workshop, we asked the participants to collectively agree on one or more solutions to implement within the screening programme. Figure 1 provides an overview of our entire approach.

Those who attended in-person interviews were given a transport reimbursement of KES 500 (US$3). We used the Consolidated Criteria for Reporting Qualitative Research (COREQ) and Strengthening the Reporting of Observational Studies in Epidemiology (STROBE) checklists to report our study (online supplemental appendix 3).

### Findings

#### Interviews

We made 143 phone calls to invite people to participate in in-person and telephone interviews. Three people declined; 29 did not pick up after three calls; and 34 people agreed but either were not home (13 people) or did not arrive at the agreed interview location (21 people); six were not eligible as they told us they had actually received care (ie, they had not been checked-in properly) and four had moved to a different part of the country. In total, we conducted 36 telephone interviews and 31 face-to-face interviews over the course of 8 days in September 2023. All our participants were aged 18–44 years old and 47.8% were female.

We ended up performing more interviews than were needed to achieve thematic saturation with the 12+2 approach due to the efficiency of our data collectors. A detailed retrospective saturation analysis, presented in online supplemental appendix 4 (table 1-4 and figure 1-2), concluded that approximately 30 interviews were required to reach thematic saturation (online supplemental appendix 4,table 1). We conducted 31 in-person interviews to enable fair comparison between telephone and in-person interviews for our embedded study.

Online supplemental table 1 (Appendix 5) present the 21 unique barriers that were identified along with all of the quotes from both sets of interviews. Direct, indirect and opportunity costs; long queues; difficulty getting time off work; and insufficient information about opening times and dates emerged as important themes. We also identified several meta-themes; participants were generally able to access the clinic locations but left after seeing long queues of several hundred people. Many felt they could not ‘waste time’ waiting to be seen, given the associated loss of potential earnings.

Another important cross-cutting theme was the perceived lack of information about the clinics: where they were, days of operation, opening and closing times, and what services were available. Assumptions around (non-existent) costs and early closures also prevented some people from attending.

I also forgot the exact location where I was to go for the eye check-up and no one followed up to remind me of the place and date. MT33, telephone

They did not tell us if we need to come with money or not. Eye treatment, we are usually told to come with money. I assumed I’ll go there and they will ask me for money and I did not have it. MFK204, in-person

One interviewee also told us that the counselling he had received at the point of referral was inadequate. He wanted more information about what would happen at the clinic and on why attending was important, especially given that he did not even realise he had a problem:

They just told me that I have problems with my eyes and I should visit [town name] dispensary so I did not know what I was going to do there, is it surgery, is it being given medication, is it being tested again? And for me I have always known that my eyes are okay, and on that day they told me that they are not okay. They were very brief and I didn't know what to expect, so that shock of being told that I have an eye problem which I have never had before is the reason why I did not go. MFI03, in-person

In terms of novel barriers, one person told us that they left the queue because they were “an introvert” and didn’t like the crowd (MT772, telephone); another felt their eye problem needed emergency treatment and sought care elsewhere (MFK02, in-person). One interviewee specifically named male health seeking behaviour as the main reason he didn’t attend:

“As a man it is very hard to prioritize my health as I am manly focused on my family’s wellbeing and It is easy to forget my health needs” (MT250Z, telephone).

Finally, one young man explained that being made to queue alongside women and children was shameful:

There were women on the line. They could have different lines for youths and women for some us to be comfortable because it is shameful to be on the same line with women and children, with worries how they will perceive me as young man. It was a challenge for me to just stand there with women… I had to go back that day without being attended even though right now my eyes have a problem. MT040, telephone

The 25 proposed solutions to improve access centred around reducing the clinic queue lengths so that people could be seen quickly and then get back to work. Ideas included adding more clinics, holding them closer to villages or workplaces, increasing staff punctuality and speed, scheduling fewer people to attend each day and extending the opening days and hours. The other meta-theme related to the provision of more detailed information around clinic services and opening times. Table 1 presents a summary of all 25 suggested solutions along with illustrative quotes. A full list of all solution-related quotes is presented in online supplemental appendix 6(table 2).

**Table 1:** Proposed solutions, feasibility review, ranking scores and workshop consensus

Proposed solution	Illustrative quote	Feasibility review	Mean score*	Workshop consensus
Costs				
Provide transport fare	*“*If I can be provided with fare, I would definitely attend.” MT09	Insufficient programme budget	—	Removed before ranking: insufficient programme budget
Subsidise treatment	“You should consider needy people who cannot afford [spectacles].’’ MT148	Treatment already free	—	Removed before ranking: treatment already provided free of charge
Pay people to attend	“I’m asking if we can be compensated with the amount we could have been paid.” MFI04	Insufficient programme budget	—	Removed before ranking: insufficient programme budget
Distance and transport				
Move clinics closer to where people live and work	“The outreach should be situated in a place which is easily accessible.” MT28	Potentially feasible	2.94	Not currently feasible with available programme resourcing
Provide transport	“If you give me transport and day to come, I will come.” MT10	Insufficient programme budget	—	Removed before ranking: insufficient programme budget
Provide door-to-door services	“Consider door-to-door services, but I don't know if that’s possible.” MFI03	Insufficient programme budget	—	Removed before ranking: insufficient programme budget
Clinical service quality				
Improve punctuality and efficiency	“Those that are providing the services should be punctual and fast.” MT07	Potentially feasible; new measures already in place	2.85	Work already underway; no further specific testable ideas identified
Ensure all medicines and glasses provided on-site	“After being screened you should receive all the services, like medicine and everything.” MFNG05	Potentially feasible	2.96	Not possible to provide comprehensive specialist services at every clinic. Communication issue around which services are available
Add more staff at each clinic	“The doctors attending people should be many.” MFK009	Potentially feasible	2.95	Not currently feasible with available programme resourcing
Facilities				
Keep clinics open for more weekdays	“Add more days for the clinic so that people who did not manage to attend can still get a chance.” MT174Z	Potentially feasible	2.91	Not currently feasible with available programme resourcing
Extend clinic opening hours (eg, until 18:00)	“If it’s possible, if the exercise could extend the working hours, let’s say from 4pm to 6pm.” MFK02	Potentially feasible	2.5	Not currently feasible with available programme resourcing
Keep clinics open on the weekend	“Maybe if the exercise could be scheduled off working days—Saturday or Sunday.” MFK02	Potentially feasible	2.56	Not currently feasible with available programme resourcing
Add more clinic locations	“Have more work stations where people can easily access services.” MT010Z	Potentially feasible	2.94	All available and appropriate spaces currently being used, including public buildings
Hold mop-up clinics for those who didn't attend	“The outreach should be conducted another time so that the people who were left unattended could be attended.” MT48	Potentially feasible	2.93	Carries cost implication; may be feasible in future if additional funds available
Give each person a specific appointment slot	“The attendance number should be issued in line with the referral messages.” MT772	Potentially feasible	2.56	Not operationally feasible
Schedule fewer people per day to reduce queues	“I would like it if you control the number of people you schedule for a particular day.” MFK01	Potentially feasible	2.83	Not feasible without additional clinic days, which carry associated costs
Awareness and communications				
Send reminder text on appointment day and day before	“A reminder message should be sent before and during that day to attend the clinic.” MT656	Potentially feasible	2.88	SELECTED. Messages already sent day before; additional message added on day of appointment
Send text to those who missed clinic to invite to another date	“If a person never made it to the outreach, he should be notified of the next clinic.” MT46	Potentially feasible	2.93	Predicated on mop-up clinics; no funds currently available
Phone reminder for those who cannot read	“Maybe you can remind us by calling us—as you have today.” MFKI08	Potentially feasible	2.93	Operationally feasible but time-consuming and expensive; to be considered if additional funds available
Explain why attending clinic is important at referral	“During screening we should be given more information why this clinic is important.” MT656	Potentially feasible	2.76	SELECTED. Can be provided verbally and in follow-on SMS reminder
Use churches and radio to remind people to attend	“Mobilize the clinical outreach, especially on churches and radios.” MT48	Potentially feasible	2.85	Risk of attracting unscreened individuals and overwhelming services
Specify clinic opening and closing times at referral	“Communicate well on the exact opening and closing times, so that people may not come and get stranded.” MFNG03	Potentially feasible	2.77	SELECTED. Can be provided verbally and in follow-on SMS reminder
Allow people to choose their appointment day	“Next time they should ask me when I am available instead of putting a date for me.” MT100	Potentially feasible	2.43	Not operationally feasible
Clarify services available and any costs at referral	“Clarify information… Anything free—people think it’s hidden things.” MT100	Potentially feasible	2.88	SELECTED. Can be provided verbally and in follow-on SMS reminder
Other				
Separate queues for younger people, women and children and elderly	“Address the line issue and have women on their [own] lines… youth can just have their line.” MT040	Potentially feasible	2.6	Separate lines for women/children and older people deemed feasible; imposing separation may cause problems for groups attending together

* Mean score from telephone ranking survey (n=401): 1=small difference, 2=moderate difference, 3=big difference. Solutions removed as completely infeasible by the implementing partner prior to ranking are shaded red and shown with a score of ‘—’. Solutions selected by the multistakeholder workshop for implementation are shaded green.

### Reviewing feasibility

As planned, we presented the list of all 25 suggestions to senior representatives from the implementing partner. We asked them to identify any suggestions that would be completely infeasible to deliver, given that they are responsible for funding all aspects of the programme. They felt that the programme budget would not permit additional payments for transport reimbursement or attendance incentives. Given that the outreach clinics involve multiple members of staff and large volumes of equipment and supplies, they also felt that it simply wouldn’t be feasible to deliver a door-to-door version of the outreach clinic. These suggestions were removed from the list. The director of Peek Vision agreed with each of these decisions. The remaining 21 suggestions were put to a representative sample of people from the left behind group in a ranking survey.

### Survey and selection workshop

It took 2 days to train the data collectors and pilot the survey, and 5 days to complete ranking exercise. In total, data collectors called 440 people, of whom 401 completed the survey (response rate=91%).

All of the suggestions received a mean score between 2.4 and 2.9, indicating that all of the potential solutions were felt to offer at least a moderate chance of improving attendance. Table 1 presents the scores for individual-level solutions (i.e. those that could be tested in an individually randomised RCT) and solutions that would be implemented at the clinic level (i.e. requiring testing with cluster RCTs).

We presented the survey findings to members of the online workshop and then facilitated a discussion around each of the proposed solutions in turn.

The majority of the suggested clinic-level interventions required additional human resources or clinic locations. The programme funder and implementing partner recognised the issues around long queue times in some locations, but were very clear that inflation had already taken the programme over budget and there were no spare resources to introduce additional clinics or staff. The top-ranked suggestion related to frustration experienced by people who attended the treatment outreach clinic but were found to have a complex eye problem that required onward referral to the local hospital for specialist spectacles (where care is subsidised but not free). The workshop participants also recognised this problem, but agreed that it was not possible to have advanced ophthalmic care services present at each outreach clinic. Workshop participants suggested clarifying the process of tiered referrals during counselling. Participants felt that organising separate queues for different ages and genders would be practically feasible; however, imposing separation may cause problems for friends/family/colleagues who attend together. After reviewing all of the suggestions, the workshop participants unanimously agreed to implement and test the following bundle of interventions relating to counselling and the provision of enhanced information in SMS reminders:

- Send a reminder text on the appointment day and the day before.
- Clarify what services are available at the outreach clinic and costs linked to these services.
- Specify clinic opening and closing times at the point of referral.
- Explain why attending the clinic is important at the point of referral.

Overall, the ethics review process took 4 months. The interviews took 9 days to complete, and the survey took 7 days, including training and piloting. We held the multistakeholder workshop 1 month after the survey had concluded, with the delay driven by scheduling challenges.

The screening programme currently sends SMS reminders on the day of referral and the day before the clinic appointment. We drafted a new verbal counselling script and SMS reminder message that included the suggested new elements that were agreed in the workshop. We asked the workshop participants to review the new wording via email, as well as two representatives from the left-behind group. Based on this feedback we made three minor changes. The original script and description of these changes is presented in online supplemental appendix 7. The enhanced counselling and SMS reminder intervention bundle was tested in a subsequent RCT, published elsewhere.^31^

### Sensitivity analysis

We conducted ten additional interviews with high-income individuals aged 18–44 who had not managed to access care, based on previous evidence that this group were potentially facing unique barriers. We triangulated the themes with those from the other interviews. High-income interviewees did not mention loss of wages as a potential barrier, but the cost of treatment and transport were raised, along with conflicting engagements, long queues and lack of clear information. No unique or discordant barriers were identified. Similarly, the solutions raised were closely aligned with those from the other interviews, including requests for financial subsidies and incentives—suggesting that this group were not necessarily affluent; that is, our original ‘high-income’ threshold (US$2600/year - aligning with the national top income tax threshold) was set too low. The only unique solutions related to; a request for more stylish spectacle frames; “Next time come with spectacles of good standard, not for the old people.” (HIG3); the use of Community Health Promoters to remind people to attend; and asking for permission from employers to get time off. Full quotes and codes are presented in online supplemental appendix 8.

## Discussion

We conducted 67 interviews and 401 solution ranking exercises with people from the sociodemographic group that had previously been found to face the greatest barriers to accessing eye care services in Meru; young adults aged 18–44 years. They told us that lack of clear information and the opportunity costs associated with long queues were the main barriers to receiving care. While lack of clear information and inadequate counselling have emerged from multiple previous studies in diverse settings and populations,^32–36^ the theme of long queues does not commonly appear in the wider literature or feature as one of the barrier options that is used in Rapid Assessment of Avoidable Blindness surveys that have been deployed in over 80 countries.^37^ We postulate that the ubiquity and fundamental intractability of this problem in resource-scarce settings leads to them being perceived as the status quo rather than a specific barrier. Research from several other African countries suggests that most patients generally wait 1–4 hours to receive ambulatory care,^38–43^ and even in a well-resourced setting like the UK, 10% of patients are currently waiting more than 12 hours to receive emergency care.^44^ Younger adults may be more sensitive to long wait times as they are more likely to be in active employment than older adults, and may be more likely to be ‘hustling’ with multiple informal jobs that do not provide protected sick leave, meaning that they experience the greatest potential opportunity costs from waiting in line for several hours.^45^

Rapidly surveying a representative sample of younger adults enabled us to move from subjective to generalisable themes. For instance, if we had stopped after completing the interviews, we would not know whether the issue of mixing men and women in the queues was a major structural barrier to access for this group, an unusual individual quirk, or something in-between. In the event, we found that scores accorded to all of the 21 potential solutions clustered between 2.5 and 3.0, indicating that members from the left behind group felt that all of the suggestions were moderately-to-highly likely to improve access to care. We cannot exclude the possibility that the universally high scores are at least partially driven by cultural norms and/or a form of social desirability bias.^46,47^ When we re-run this approach in other settings we decided that introducing a small number of dummy (i.e. deliberately bad) suggestions during the intervention ranking survey would help to tease this apart.

The final set of interventions primarily focus on empowering individuals with information. However, a different perspective is that the workshop participants dismissed service-side solutions in lieu of minor interventions that place responsibility for access on intended service users, in line with the widely debunked ‘information deficit model’.^48^ Allen has previously argued that efforts to equitably advance UHC and ‘health for all’ should focus on unjust social structures and resist focusing wholly on ‘downstream’ technocratic solutions.^3^ In the context of this study, the paucity of good jobs, the absence of adequate social welfare systems and strong labour laws that guarantee paid sick leave are examples of major structural challenges for younger adults; however, these were not raised by the interviewees. Our feasibility screening step, while necessary to identify actionable service modifications, means that many of these structural barriers, such as inadequate social protection, absence of paid sick leave and gender norms, were documented but not addressed within this programme. These findings are relevant to policy actors operating at a different level (national government, labour regulation, social welfare) and we encourage readers to consider the full list of identified barriers in online supplemental appendix 5 in this broader context. Culturally sensitive adaptations to service delivery, including gender-sensitive queue management and targeted public messaging, warrant further investigation.

Mapping our findings onto the Levesque and Obrist frameworks, barriers and proposed solutions were concentrated in three dimensions: availability and accommodation (queue length, opening hours, clinic days and locations); affordability in its broadest sense (opportunity costs, loss of earnings and transport costs); and approachability and awareness (information deficits at the point of referral). The adequacy and acceptability dimensions were raised but were judged by workshop participants to lie beyond the immediate scope of programme modification. Obrist *et al*’s emphasis on livelihood insecurity is directly reflected in our data: the dominant barrier was not distance or cost of care per se, but the opportunity cost of queuing, which is a function of precarious employment rather than a modifiable programme characteristic. This is a critical finding for programme designers: interventions that reduce wait times address a structural issue for low-income groups rather than a logistical inconvenience.

## Limitations

This study has a number of limitations. First, 29 of 143 individuals (20%) we attempted to contact for interview did not answer after three call attempts. Those hardest to reach by telephone are plausibly those facing the greatest time constraints. However, opportunity costs and queue-related barriers were already the dominant themes among those we did interview, so a more complete sample would be unlikely to have yielded categorically different findings. Second, social desirability bias may have inflated ranking scores in the telephone survey, contributing to the narrow high clustering of scores between 2.4 and 2.9; this limits the discriminatory power of the ranking exercise as a tool for prioritisation. Third, while our feasibility screening step was necessary to identify actionable service modifications, it meant that structural barriers were documented but not addressed. The specific barriers and solutions identified are context-dependent; however, community-based screening and outreach models are common across sub-Saharan Africa and South Asia, and the thematic findings are likely to resonate in similar programmes. Crucially, our methodological approach was explicitly designed for transferability and could be applied by any community screening programme to generate locally valid, actionable evidence within a matter of weeks.

The greatest strengths and limitations of our approach pertain to voice and epistemic justice.^13^ We strove to centre our approach around the perspectives of those with lived experience of not being able to access care. The ‘pose’ and ‘gaze’ of the project^15^ focus on the credibility and value of this group, as well as locally recruited data collectors’ work in sensemaking through their initial interpretative work as they engaged with the analytic matrix. Findings from this process fed directly into the local programme, with their implications wholly determined by local stakeholders. Although Europeans and Kenyan public health experts were involved in leading the project, the primary interpretation was locally owned and the resulting solutions were locally implemented for the benefit of local Meru residents.

The programme funder and implementing partner held veto rights as they were ultimately responsible for delivering service modifications and could not be ‘made’ to implement any of the suggested service modifications. However, community, charity and public health stakeholders reached one accord with the programme leads on the infeasibility of introducing additional staff of clinics given their shared appreciation of how inflation had decimated the programme budget. Nevertheless, our approach involved (and necessitated) epistemic tension; even though all of the solutions came from the left-behind group, the final decision on how to modify the programme required consensus between different stakeholder groups with imperfectly overlapping interests and unequal power in the final determination. We feel that including programme managers is a design strength rather than a flaw, as there is no value in presenting programmes with a *post hoc* shortlist of unworkable suggestions. Furthermore, the process of bringing them into conversation with community members, eye care advocates and representatives from the left behind group can potentially create shared understanding of the problems and initiate constructive dialogue. Our role as ‘external’ research leads primarily involved developing rapid and robust methods that would yield non-tokenistic and robust interventions, as well as convening actors and facilitating the overall process. We aim to work with local partners to embed this approach so that future iterations can be wholly led by local teams.

Our unplanned sampling deviation led us to conduct more interviews than expected. The post hoc sensitivity analysis presented in online supplemental appendix 3 suggests that if we had adhered to our original ‘12+2’ approach, we would have missed four barriers and 12 additional solutions. We agree with Guest *et al* that current approaches to assessing and reporting saturation in qualitative research are opaque; however, our empirical analysis suggests that in studies like ours, saturation is only reached after three interviews in a row reveal no new themes, after a minimum of 15 initial interviews have already been conducted (ie, a ‘15+3’ approach).^21^ This is illustrated in Figure 2.

## Conclusions

Interviews and ranking surveys conducted with younger adults who were not able to access eye care services identified 21 barriers and 25 potential solutions, centring around reducing the time it takes to be seen and providing clearer information about local services. Multistakeholder workshop participants ended up selecting interventions that did not fundamentally alter how clinics are operated, instead opting for amendments to the way that information is provided. Even so, these service modifications came directly from those who were being left behind and were rated as moderately-to-highly likely to improve access to care. Including service funders and managers in interpretive and deliberative processes means that our findings resulted in concrete, locally led service modifications. A subsequent embedded, pragmatic RCT found that these modification increased attendance from 32.1% to 39.0% among young adults in Meru.^31^

## Supplementary material

10.1136/bmjph-2025-002856 online supplemental file 1

## Data Availability

All data relevant to the study are included in the article or uploaded as supplementary information.
