# Supplementary material for: Identifying barriers and potential solutions to improve equitable access to community eye services in central Kenya: a rapid exploratory sequential mixed methods study
Source: BMJ Public Health. 2026 Sep 21;4(3):e002856. doi: 10.1136/bmjph-2025-002856 (PMC13599916; doi:10.1136/bmjph-2025-002856)
Supplement: Supplementary file 1 [file bmjph-4-3-s001.docx]

Appendix 1: Call scripts

Telephone interview consent script and topic guide

Hello, my name is___________. I am a researcher from KEMRI, working with the Ministriy of Health and the VIP eye screening programme.

Your recently had your eyes screened and were found to need further assessment. Our records indicate that, like many other people, you were unable to attend that appointment.

You are being contacted because you have previously provided consent to be contacted regarding research being conducted for eye care services. I am calling to invite you to participate in a 15-minute interview. Your participation is completely voluntary. This means that you do not have to do it unless you want to.

We want to understand the barriers that prevented you from attending. We are also asking about how we could change the VIP programme to make it easier for people like you to attend appointments.

Before agreeing, here is the background information that you need to know:

We have invited you because, like many other people, you did not attend. We want to hear about the issues that you personally faced that prevented you from attending, and your ideas on how to make things easier. In total we are aiming to interview about 20 people.

Who are we? I work with a group of researchers from the KEMRI and the London School of Hygiene and Tropical Medicine. We are working to improve the VIP eye screening programme. The leaders of the research are Prof Michael Gichangi and Dr Luke Allen.

We will take the responses from all of the interviews and discuss the ideas for improvement with the leaders of the national programme. We hope to use your suggestions to make the programme work better.

We are also conducting a set of face-to-face interviews with other people who did not manage to access care. We want to compare the responses we get from these different approaches.

In this 15-minute interview there are no risks or benefits to you. It is important to note that agreeing or declining to take part does not have any impact on the services you receive.

You can stop the interview at any time.

I will record the interview. Our team will anonymise your data and keep it safe and secure on a password-protected computer in London. When the study is completed, we will write-up our findings and publish them online so that other researchers can use the information to help people in other places.

The KEMRI and London School of Hygiene and Tropical Medicine ethics committees have both approved this study.

You can ask me any questions you like now. I can also give you the email address and phone number of the lead researchers if you’d like to contact them directly [provide the contact details for Sarah Karanja or Luke Allen as required]. If you have any other concerns I can also give you the contact details for the London School of Hygiene and Tropical Medicine Research Governance and Integrity Office.

Do you have any questions?

Are you happy to begin the interview?

[If yes, proceed; if no thank them for their time and end the call]

**Opening questions**

To start with, during the home visit by the vision impact project team, please describe your experience with the eye screening.

Probes:

- How long did the examination take?
- Was the test comfortable? If yes, why? If no, why not?

**Barrier elicitation questions**

You were assessed at home and found to have an eye problem that required further assessment. You were asked to attend the community eye clinic on [date] and reminders were sent to this mobile number.

- In your own words, can you talk me through why we didn’t see you at that clinic?

**Probing questions**

- Are there any other factors that prevented you from attending?
- Is there anything else you’d like to share?
- Of the issues you mentioned, which is the most important?

**Solution elicitation questions**

The last part of the interview is exploring whether there is anything we could do to address these barriers and make it more likely that you will attend in the future.

- So to start, what would make the biggest difference?

**Probing questions**

- What else would help?
- What other changes could we make to the programme that would make it easier for you to attend?
- Are there any other specific changes that we could make to the way that the programme or eye clinics run?
- You mentioned [list their proposed solutions]. Some of these may be beyond our control, but if we managed to [list their proposed programme-related changes], do you think that would be enough?

That’s the end of my questions. Is there anything else you would like to add?

**Probing questions**

- After missing the outreach clinic appointment, did you seek medical care from a different healthcare facility for your eye condition?
- If no, why not?
- If yes, where did you seek medical care?

Thank you so much for your time.

Call script to invite people to participate in an in-person interview.

Hello, my name is___________. I am a researcher from the Kenya Medical Research Institute (KEMRI), working with the Ministries of Health and Education on the Vision Impact Project eye screening programme.

You recently had your eyes screened at the community outreach programme and was found to need further assessment. Our records indicate that, like many other people, you were unable to attend that appointment.

You are being contacted because you have previously provided consent to be contacted by Ministry of Health partner organisations regarding research being conducted for eye care services. I am calling to invite you to participate in an in-person interview in the next few weeks. Your participation is completely voluntary. This means that you do not have to do it unless you want to.

If you agree, I will arrange to meet you in or near where you live to ask you some questions about the barriers that prevented your child from attending. I also want to ask about how we could change the Vision Impact Project to make it easier for people to attend appointments.

To compensate you for your time you will receive a [KES 500 equivalent] airtime voucher.

If you are interested, I can send you the full study information via WhatsApp or email or talk it through on the phone with you now.

[switch to phone PIL script here as required]

Appendix 2: Quantitative Ranking Survey

Interviewer name

Short-answer text

Participant ID

Short-answer text

**Consent**

Good morning/afternoon

My name is .... and I’m calling from the Vision Impact Project eye screening programme. We saw you a few weeks ago and referred you to the local clinic, but we did not see you on your appointed day.

In fact, half of all people who were referred did not attend. We have sought feedback on ways we could improve our service, and I wanted to ask you which of the ideas we have stand the best chance of helping people like you to access care. It should take approximately 15 minutes of your time.

If you are happy to proceed, I need to tell you a bit more about the survey. I will then double-check that you are still happy to proceed.

I will ask you about a set of potential changes that we are thinking about making. I will ask you to rate each one in terms of how likely you think it is to make a difference at helping people access our clinics.

Your responses will help us to shape and improve our services for others, but there are no direct benefits to you for taking part. Thinking about the issues that prevented you from getting care may be distressing to you. If you face any discomfort because of the questions asked, you can skip any question or ask to end the call whenever you choose.

If you don’t want to take part, that’s ok. You can drop out of the survey at any point. Your decision will not affect your health care or your future relations with the Vision Impact Project in any way.

Your anonymised answers will be combined with those from other people and kept safe and secure on password-protected computers in Nairobi and London. None of the data will be used for commercial use. We will publish our findings in a research journal and in a public repository so that other researchers can learn from what we find. You personal information will not be included in our findings and there is no way that you can be identified from any of the reports that we will produce.

If you have any questions, you can ask me now, or I can put you in contact with the study coordinator -  Sarah Karanja from Kenya Medical Research Centre. If you have any questions about your rights as a research participant, I can connect you with the Kenya Medical Research Centre Ethics team who approved this survey.

Does that all make sense? Do you have any questions for me?

Are you happy for me to start?

**The first two potential improvements are aimed at making it easier for people to get to a clinic.**

There are three options to choose from:

1. It would make a small difference - i.e. it might help a few people, but the impact is likely to be minimal

2. It would make a moderate difference - i.e. it would greatly increase the chances, but it would not be enough by itself to guarantee attendance by itself

3. It would make a big difference - i.e. if we introduced this change then you or people like you would definitely attend

If we add more clinic locations to reduce the distance people have to travel, how likely is that to make a difference?

1 []

2 []

3 []

Expand the list of referral clinics to include additional public health facilities and private and faith-based hospitals

1 []

2 []

3 []

**The next set of improvements are about reminding people about the clinic**

Explain why attending clinic is important at the point of referral

1 []

2 []

3 []

Clarify what services are available at the outreach clinic and specify if there are any costs linked to these services

1 []

2 []

3 []

Send a reminder text on the appointment day and the day before

1 []

2 []

3 []

Send a text to individuals who missed the outreach clinic to invite them to attend on another day

1 []

2 []

3 []

Phone people who cannot read to remind them to attend

1 []

2 []

3 []

Use churches and radio broadcasts to remind people to attend

1 []

2 []

3 []

**The next set of improvements are about extending clinic opening hours**

 Keep the clinics open for a greater number of weekdays

1 []

2 []

3 []

Specify clinic opening and closing time at the point of referral

1 []

2 []

3 []

Keep the clinics open until 6pm

1 []

2 []

3 []

Keep the clinics open on the weekend

1 []

2 []

3 []

Hold mop-up clinics a week or so later for those who didn’t manage to attend

1 []

2 []

3 []

Allow people to choose their appointment day

1 []

2 []

3 []

**The next set of improvements are about reducing waiting times**

Add more staff to reduce the queue waiting time

1 []

2 []

3 []

Refer fewer people for the same day to reduce queue numbers on each day

1 []

2 []

3 []

Give each person a specific appointment slot

1 []

2 []

3 []

Increase the punctuality and speed at which our staff work

1 []

2 []

3 []

Have a separate line for younger people

1 []

2 []

3 []

Have separate lines for women and children

1 []

2 []

3 []

Have a separate line for older people

1 []

2 []

3 []

**The final set of improvements are about stocks & supplies**

Ensure that clinics are fully stocked so that all medicines and glasses can be provided on-site

1 []

2 []

3 []

 Thank you so much for your time, we really appreciate your generosity, your responses will help us to improve the service

Is there anything else you would like to feed-back to us?

Long-answer text

Thank you again, goodbye

Appendix 3: COREQ and STROBE checklists

STROBE Statement—checklist of items that should be included in reports of observational studies

|  | **Item No.** | **Recommendation** | **Page  No.** | **Relevant text from manuscript** |
| --- | --- | --- | --- | --- |
| **Title and abstract** | 1 | (*a*) Indicate the study’s design with a commonly used term in the title or the abstract | 1 | ‘ A rapid exploratory sequential mixed methods study’ |
|  |  | (*b*) Provide in the abstract an informative and balanced summary of what was done and what was found | 2 |  |
| **Introduction** | | | |  |
| Background/rationale | 2 | Explain the scientific background and rationale for the investigation being reported | 3 |  |
| Objectives | 3 | State specific objectives, including any prespecified hypotheses | 3-4 |  |
| **Methods** | | | |  |
| Study design | 4 | Present key elements of study design early in the paper | 5-6 |  |
| Setting | 5 | Describe the setting, locations, and relevant dates, including periods of recruitment, exposure, follow-up, and data collection | 4 |  |
| Participants | 6 | (*a*) *Cohort study*—Give the eligibility criteria, and the sources and methods of selection of participants. Describe methods of follow-up  *Case-control study*—Give the eligibility criteria, and the sources and methods of case ascertainment and control selection. Give the rationale for the choice of cases and controls  *Cross-sectional study*—Give the eligibility criteria, and the sources and methods of selection of participants | 4,6 |  |
|  |  | (*b*) *Cohort study*—For matched studies, give matching criteria and number of exposed and unexposed  *Case-control study*—For matched studies, give matching criteria and the number of controls per case | N/A |  |
| Variables | 7 | Clearly define all outcomes, exposures, predictors, potential confounders, and effect modifiers. Give diagnostic criteria, if applicable | 7,8,9 |  |
| Data sources/ measurement | 8* | For each variable of interest, give sources of data and details of methods of assessment (measurement). Describe comparability of assessment methods if there is more than one group | 7-9 |  |
| Bias | 9 | Describe any efforts to address potential sources of bias | 19,20 |  |
| Study size | 10 | Explain how the study size was arrived at | 6,9,10 |  |

Continued on next page

| Quantitative variables | 11 | Explain how quantitative variables were handled in the analyses. If applicable, describe which groupings were chosen and why | 9 |  |
| --- | --- | --- | --- | --- |
| Statistical methods | 12 | (*a*) Describe all statistical methods, including those used to control for confounding | 9 |  |
|  |  | (*b*) Describe any methods used to examine subgroups and interactions | 8 |  |
|  |  | (*c*) Explain how missing data were addressed | N/A |  |
|  |  | (*d*) *Cohort study*—If applicable, explain how loss to follow-up was addressed  *Case-control study*—If applicable, explain how matching of cases and controls was addressed  *Cross-sectional study*—If applicable, describe analytical methods taking account of sampling strategy | N/A |  |
|  |  | (*e*) Describe any sensitivity analyses | N/A |  |
| **Results** | | | | |
| Participants | 13* | (a) Report numbers of individuals at each stage of study—eg numbers potentially eligible, examined for eligibility, confirmed eligible, included in the study, completing follow-up, and analysed | 13 |  |
|  |  | (b) Give reasons for non-participation at each stage | 13 |  |
|  |  | (c) Consider use of a flow diagram | N/A |  |
| Descriptive data | 14* | (a) Give characteristics of study participants (eg demographic, clinical, social) and information on exposures and potential confounders | 13 |  |
|  |  | (b) Indicate number of participants with missing data for each variable of interest | N/A |  |
|  |  | (c) *Cohort study*—Summarise follow-up time (eg, average and total amount) | N/A |  |
| Outcome data | 15* | *Cohort study*—Report numbers of outcome events or summary measures over time | N/A |  |
|  |  | *Case-control study—*Report numbers in each exposure category, or summary measures of exposure | N/A |  |
|  |  | *Cross-sectional study—*Report numbers of outcome events or summary measures | 10-18 |  |
| Main results | 16 | (*a*) Give unadjusted estimates and, if applicable, confounder-adjusted estimates and their precision (eg, 95% confidence interval). Make clear which confounders were adjusted for and why they were included | N/A |  |
|  |  | (*b*) Report category boundaries when continuous variables were categorized | N/A |  |
|  |  | (*c*) If relevant, consider translating estimates of relative risk into absolute risk for a meaningful time period | N/A |  |

Continued on next page

| Other analyses | 17 | Report other analyses done—eg analyses of subgroups and interactions, and sensitivity analyses | 18 |  |
| --- | --- | --- | --- | --- |
| **Discussion** | | | | |
| Key results | 18 | Summarise key results with reference to study objectives | 18-19 |  |
| Limitations | 19 | Discuss limitations of the study, taking into account sources of potential bias or imprecision. Discuss both direction and magnitude of any potential bias | 20 |  |
| Interpretation | 20 | Give a cautious overall interpretation of results considering objectives, limitations, multiplicity of analyses, results from similar studies, and other relevant evidence | 22 |  |
| Generalisability | 21 | Discuss the generalisability (external validity) of the study results | 4,19 |  |
| **Other information** | |  | | |
| Funding | 22 | Give the source of funding and the role of the funders for the present study and, if applicable, for the original study on which the present article is based | 23 |  |

*Give information separately for cases and controls in case-control studies and, if applicable, for exposed and unexposed groups in cohort and cross-sectional studies.

**Note:** An Explanation and Elaboration article discusses each checklist item and gives methodological background and published examples of transparent reporting. The STROBE checklist is best used in conjunction with this article (freely available on the Web sites of PLoS Medicine at http://www.plosmedicine.org/, Annals of Internal Medicine at http://www.annals.org/, and Epidemiology at http://www.epidem.com/). Information on the STROBE Initiative is available at www.strobe-statement.org

Appendix 4: Post-hoc saturation analysis

We originally set out to use Guest and colleague’s simple formula for assessing whether thematic saturation had been achieved [1]. They describe saturation as “the point in data collection and analysis when new incoming data produces little or no new information to address the research question”, and identify three components that can be used to establish when this point occurs:

- The base, i.e. the minimum number of interviews to be conducted before calculating whether saturation has been achieved. It is similar to the ‘initial analysis sample’ described by Francis et al.[2] Guest et al suggest base sizes of 4-6, based on a review of the literature.
- The run length, i.e. the number of interviews that we review to assess whether any new themes have emerged, with reference to those that have already emerged in the base. Guest et al suggest conducting runs of 2-3.
- The new information threshold, i.e. the number of new themes identified in each run as a proportion of the themes identified in the base interviews. Guest et al suggest that researchers may want to stipulate that saturation is only achieved when a run identifies no new themes (new information threshold = 0%), or that the new themes identified represent ≤5% of the themes identified in the base interviews.

We set out to use a conservatively large base size of 12 interviews, based on a literature review presented in our protocol,[ref] with runs of two, and a 0% new information threshold. We also decided that we would compare the new information (number of themes) identified in the runs against all previous interviews, rather than just those in the base.

As detailed in our findings section, we ended up conducting 36 interviews. By reviewing the number of themes that were identified in each interview, we are able to retrospectively assess the point at which saturation would have been reached using a range of different base, run, and new information threshold permutations.

For our initial analysis (Table 1), we used a base of 12 and compared new information obtained from each run of two interviews against the themes identified from the original base of 12 interviews. We performed individual analyses for barriers and solutions. We highlighted the points at which the new of 0% was met.

**Table 1: Saturation for barriers and solutions with runs of two compared to a base of 12**

| **Interview number** | **Barriers** | | | | **Solutions** | | | |
| --- | --- | --- | --- | --- | --- | --- | --- | --- |
|  | Barriers identified | Novel barriers | Cumulative barriers | New info: runs of 2 | Solutions identified | Novel solutions | Cumulative solutions | New info: runs of 2 |
| **1** | 4 | 4 | 4 | N/A | 3 | 3 | 3 | N/A |
| **2** | 4 | 1 | 5 | N/A | 3 | 2 | 5 | N/A |
| **3** | 3 | 3 | 8 | N/A | 1 | 1 | 6 | N/A |
| **4** | 2 | 0 | 8 | N/A | 1 | 1 | 6 | N/A |
| **5** | 3 | 2 | 10 | N/A | 2 | 0 | 6 | N/A |
| **6** | 1 | 0 | 10 | N/A | 2 | 1 | 7 | N/A |
| **7** | 1 | 1 | 11 | N/A | 2 | 1 | 8 | N/A |
| **8** | 1 | 0 | 11 | N/A | 1 | 0 | 8 | N/A |
| **9** | 1 | 0 | 11 | N/A | 0 | 0 | 8 | N/A |
| **10** | 1 | 0 | 11 | N/A | 1 | 0 | 8 | N/A |
| **11** | 3 | 0 | 11 | N/A | 1 | 0 | 8 | N/A |
| **12** | 1 | 0 | 11 | N/A | 1 | 2 | 10 | N/A |
| **13** | 1 | 0 | 11 | N/A | 1 | 1 | 11 | N/A |
| **14** | 1 | 0 | 11 | 0.0% | 1 | 0 | 11 | 10.0% |
| **15** | 1 | 0 | 11 | 0.0% | 2 | 0 | 11 | 0.0% |
| **16** | 1 | 0 | 11 | 0.0% | 0 | 0 | 11 | 0.0% |
| **17** | 1 | 0 | 11 | 0.0% | 2 | 2 | 13 | 20.0% |
| **18** | 1 | 0 | 11 | 0.0% | 1 | 0 | 13 | 20.0% |
| **19** | 1 | 0 | 11 | 0.0% | 1 | 0 | 13 | 0.0% |
| **20** | 2 | 0 | 11 | 0.0% | 2 | 1 | 14 | 10.0% |
| **21** | 1 | 0 | 11 | 0.0% | 1 | 0 | 14 | 10.0% |
| **22** | 1 | 0 | 11 | 0.0% | 4 | 2 | 16 | 20.0% |
| **23** | 2 | 1 | 12 | 9.1% | 2 | 1 | 17 | 30.0% |
| **24** | 1 | 0 | 12 | 9.1% | 3 | 2 | 19 | 30.0% |
| **25** | 2 | 0 | 12 | 0.0% | 3 | 1 | 20 | 30.0% |
| **26** | 0 | 0 | 12 | 0.0% | 1 | 0 | 20 | 10.0% |
| **27** | 1 | 1 | 13 | 9.1% | 2 | 1 | 21 | 10.0% |
| **28** | 2 | 1 | 14 | 18.2% | 1 | 1 | 22 | 20.0% |
| **29** | 2 | 0 | 14 | 9.1% | 2 | 0 | 22 | 10.0% |
| **30** | 1 | 0 | 14 | 0.0% | 1 | 0 | 22 | 0.0% |
| **31** | 2 | 0 | 14 | 0.0% | 2 | 0 | 22 | 0.0% |
| **32** | 2 | 0 | 14 | 0.0% | 2 | 0 | 22 | 0.0% |
| **33** | 2 | 1 | 15 | 9.1% | 1 | 0 | 22 | 0.0% |
| **34** | 0 | 0 | 15 | 9.1% | 1 | 1 | 23 | 10.0% |
| **35** | 0 | 0 | 15 | 0.0% | 1 | 0 | 23 | 10.0% |
| **36** | 0 | 0 | 15 | 0.0% | 1 | 0 | 23 | 0.0% |

Table 1 shows that two interviews in a row were completed with no new barriers identified by interview number 14. By this point, 11 barriers had been identified. In the remaining 22 interviews a further four barriers were identified.

For the solutions, saturation was achieved by interview number 15. By this point 11 unique solutions had been identified. By the end of 36 interviews, a further 12 solutions had been identified.

In summary, had we used Guest and colleagues approach with a conservative base of 12, less conservative runs of two, and a very conservative new information threshold of 0%, we would have stopped after interview number 15, missing out on a third of the unique barriers that emerged across our 36 telephone interviews.

In our next analysis, we counted the total number of barriers *and* solutions (themes) that emerged from each interview. We ran six additional scenarios, using runs of two, three, and four against the base of all themes identified in the first 12 interviews. We also used runs of two, three, and four against all themes identified in all preceding interviews.

**Table 2: Different run lengths and base sizes to assess saturation, using all themes in each interview**

| **Interview number** | **Themes identified** | **Novel themes** | **Cumulative themes** | **Runs of 2** | | **Runs of 3** | | **Runs of 4** | |
| --- | --- | --- | --- | --- | --- | --- | --- | --- | --- |
|  |  |  |  | Base = 12 | Base ≥12 | Base = 12 | Base ≥12 | Base = 12 | Base ≥12 |
| **1** | 7 | 7 | 7 |  |  |  |  |  |  |
| **2** | 7 | 3 | 10 |  |  |  |  |  |  |
| **3** | 4 | 4 | 14 |  |  |  |  |  |  |
| **4** | 3 | 1 | 15 |  |  |  |  |  |  |
| **5** | 5 | 2 | 17 |  |  |  |  |  |  |
| **6** | 3 | 1 | 18 |  |  |  |  |  |  |
| **7** | 3 | 2 | 20 |  |  |  |  |  |  |
| **8** | 2 | 0 | 20 |  |  |  |  |  |  |
| **9** | 1 | 0 | 20 |  |  |  |  |  |  |
| **10** | 2 | 0 | 20 |  |  |  |  |  |  |
| **11** | 4 | 0 | 20 |  |  |  |  |  |  |
| **12** | 2 | 2 | 22 |  |  |  |  |  |  |
| **13** | 2 | 1 | 23 |  |  |  |  |  |  |
| **14** | 2 | 0 | 23 | 4.5% | 4.5% |  |  |  |  |
| **15** | 3 | 0 | 23 | 0.0% | 0.0% | 4.5% | 4.5% |  |  |
| **16** | 1 | 0 | 23 | 0.0% | 0.0% | 0.0% | 0.0% | 4.5% | 4.5% |
| **17** | 3 | 2 | 25 | 9.1% | 8.7% | 9.1% | 8.7% | 9.1% | 8.7% |
| **18** | 2 | 0 | 25 | 9.1% | 8.7% | 9.1% | 8.7% | 9.1% | 8.7% |
| **19** | 2 | 0 | 25 | 0.0% | 0.0% | 9.1% | 8.7% | 9.1% | 8.7% |
| **20** | 4 | 1 | 26 | 4.5% | 4.0% | 4.5% | 4.0% | 13.6% | 13.0% |
| **21** | 2 | 0 | 26 | 4.5% | 4.0% | 4.5% | 4.0% | 4.5% | 4.0% |
| **22** | 5 | 2 | 28 | 9.1% | 7.7% | 13.6% | 12.0% | 13.6% | 12.0% |
| **23** | 4 | 2 | 30 | 18.2% | 15.4% | 18.2% | 15.4% | 22.7% | 20.0% |
| **24** | 4 | 2 | 32 | 18.2% | 14.3% | 27.3% | 23.1% | 27.3% | 23.1% |
| **25** | 5 | 1 | 33 | 13.6% | 10.0% | 22.7% | 17.9% | 31.8% | 26.9% |
| **26** | 1 | 0 | 33 | 4.5% | 3.1% | 13.6% | 10.0% | 22.7% | 17.9% |
| **27** | 3 | 2 | 35 | 9.1% | 6.1% | 13.6% | 9.4% | 22.7% | 16.7% |
| **28** | 3 | 2 | 37 | 18.2% | 12.1% | 18.2% | 12.1% | 22.7% | 15.6% |
| **29** | 4 | 0 | 37 | 9.1% | 5.7% | 18.2% | 12.1% | 18.2% | 12.1% |
| **30** | 2 | 0 | 37 | 0.0% | 0.0% | 9.1% | 5.7% | 18.2% | 12.1% |
| **31** | 4 | 0 | 37 | 0.0% | 0.0% | 0.0% | 0.0% | 9.1% | 5.7% |
| **32** | 4 | 0 | 37 | 0.0% | 0.0% | 0.0% | 0.0% | 0.0% | 0.0% |
| **33** | 3 | 1 | 38 | 4.5% | 2.7% | 4.5% | 2.7% | 4.5% | 2.7% |
| **34** | 1 | 1 | 39 | 9.1% | 5.4% | 9.1% | 5.4% | 9.1% | 5.4% |
| **35** | 1 | 0 | 39 | 4.5% | 2.6% | 9.1% | 5.4% | 9.1% | 5.4% |
| **36** | 1 | 0 | 39 | 0.0% | 0.0% | 4.5% | 2.6% | 9.1% | 5.4% |

Table 2 shows that using a more conservative base that includes all interview conducted does not materially change the point at which saturation is deemed to have been achieved. However, increasing the run length from three to four was a associated with a much later saturation point; 32 interviews with a 0% new information threshold. Interestingly, the use of a higher new information threshold (<5%) would lead to very similar saturation points of between 14-16 interviews depending on run length.

Finally, we plotted the cumulative themes that emerged over the course of the 36 interviews. Figure 1 illustrates the fact that the majority of unique barriers were identified by interview number seven, followed by a long plateau. Shorter plateaus in the number of solutions arising from interviews 7-11 and 13-16 were followed by incremental gains until levelling off again around interview number 28. The paucity of new information arising from interviews 13-16 drive the saturation decisions in the analyses performed above.

**Figure 1: Accumulation of themes as the interviews progressed**

Cululative themes

| Interview number | All thems | Solutions | Barriers |
| --- | --- | --- | --- |
| 1 | 7 | 3 | 4 |
| 2 | 10 | 5 | 5 |
| 3 | 14 | 6 | 8 |
| 4 | 15 | 6 | 8 |
| 5 | 17 | 6 | 10 |
| 6 | 18 | 7 | 10 |
| 7 | 20 | 8 | 11 |
| 8 | 20 | 8 | 11 |
| 9 | 20 | 8 | 11 |
| 10 | 20 | 8 | 11 |
| 11 | 20 | 8 | 11 |
| 12 | 22 | 10 | 11 |
| 13 | 23 | 11 | 11 |
| 14 | 23 | 11 | 11 |
| 15 | 23 | 11 | 11 |
| 16 | 23 | 11 | 11 |
| 17 | 25 | 13 | 11 |
| 18 | 25 | 13 | 11 |
| 19 | 25 | 13 | 11 |
| 20 | 26 | 14 | 11 |
| 21 | 26 | 14 | 11 |
| 22 | 28 | 16 | 11 |
| 23 | 30 | 17 | 12 |
| 24 | 32 | 19 | 12 |
| 25 | 33 | 20 | 12 |
| 26 | 33 | 20 | 12 |
| 27 | 35 | 21 | 13 |
| 28 | 37 | 22 | 14 |
| 29 | 37 | 22 | 14 |
| 30 | 37 | 22 | 14 |
| 31 | 37 | 22 | 14 |
| 32 | 37 | 22 | 14 |
| 33 | 38 | 22 | 15 |
| 34 | 39 | 23 | 15 |
| 35 | 39 | 23 | 15 |
| 36 | 39 | 23 | 15 |

Figure 1 suggests that for this specific set of interviews, a base size of 15 and a run length of three might have yielded a more appropriate saturation point, given that new themes continued to accrue until around interview number 28. Table 3 shows that using a conservative base of ≥15 interviews and a new information threshold of 0% leads to saturation by interview number 30, 31, and 32 with respective run lengths of two, three, and four.

Researchers like Coenen et al. have suggested that run lengths of two are adequate,[3] whilst others have recommended runs of three.[2,4] Based on our findings presented in table we decided that we would use a base of ≥15 and runs of three for future studies.

**Table 3: Re-running the saturation analysis with a base of => 15 and runs of 2-4.**

| **Interview number** | **New information with a base of** ≥**15** | | |
| --- | --- | --- | --- |
|  | ***Runs of 2*** | ***Runs of 3*** | ***Runs of 4*** |
| **15** |  |  |  |
| **16** |  |  |  |
| **17** | 8.7% |  |  |
| **18** | 8.7% | 8.7% |  |
| **19** | 0.0% | 8.7% | 8.7% |
| **20** | 4.0% | 4.0% | 13.0% |
| **21** | 4.0% | 4.0% | 4.0% |
| **22** | 7.7% | 12.0% | 12.0% |
| **23** | 15.4% | 15.4% | 20.0% |
| **24** | 14.3% | 23.1% | 23.1% |
| **25** | 10.0% | 17.9% | 26.9% |
| **26** | 3.1% | 10.0% | 17.9% |
| **27** | 6.1% | 9.4% | 16.7% |
| **28** | 12.1% | 12.1% | 15.6% |
| **29** | 5.7% | 12.1% | 12.1% |
| **30** | 0.0% | 5.7% | 12.1% |
| **31** | 0.0% | 0.0% | 5.7% |
| **32** | 0.0% | 0.0% | 0.0% |
| **33** | 2.7% | 2.7% | 2.7% |
| **34** | 5.4% | 5.4% | 5.4% |
| **35** | 2.6% | 5.4% | 5.4% |
| **36** | 0.0% | 2.6% | 5.4% |

**Application to second data set**

As we had conducted a second set of 31 in-person interviews, we were able to test whether the ≥15+3 approach would have ended data collection at an appropriate point, i.e. without missing additional themes that arose in further interviews, nor prolonging data collection long after all themes had been identified.

Table 4 shows that using this approach identifies interview number 24 as the point at which saturation is achieved.

**Table 4: Saturation analysis of 31 in-person interviews using a base of ≥15 and a run length of three**

| **Interview number** | **Solutions** | **Barriers** | **All themes** | **New information** |
| --- | --- | --- | --- | --- |
| 15 | 10 | 18 | 28 |  |
| 16 | 11 | 18 | 29 |  |
| 17 | 11 | 18 | 29 |  |
| 18 | 12 | 19 | 31 | 5.6% |
| 19 | 13 | 19 | 32 | 5.6% |
| 20 | 14 | 19 | 33 | 5.6% |
| 21 | 14 | 21 | 35 | 10.5% |
| 22 | 14 | 21 | 35 | 10.5% |
| 23 | 14 | 21 | 35 | 10.5% |
| 24 | 15 | 21 | 36 | 0.0% |
| 25 | 15 | 22 | 37 | 4.8% |
| 26 | 15 | 22 | 37 | 4.8% |
| 27 | 15 | 22 | 37 | 4.8% |
| 28 | 15 | 22 | 37 | 0.0% |
| 29 | 15 | 22 | 37 | 0.0% |
| 30 | 15 | 22 | 37 | 0.0% |
| 31 | 15 | 22 | 37 | 0.0% |

Using this cutoff would have led to the inclusion of all solutions, and all but one of the barriers identified across the 31 interviews. Figure 2 plots the saturation point on a line graph.

**Figure 2: Accumulation of themes as the interviews progressed**

Based on this retrospective analysis of our data, our group has decided to use the 15+3 approach to assess saturation in our future qualitative research projects.

**Contributors**

LNA and MG are the study co-PIs. AB is the chief investigator. All authors jointly conceived the study, and analyses. EM, DG, PK, EM, LM, LK, FK and BN conducted the interviews. LNA and SK coordinated training and supervision of data collection, and led the analyses. All authors contributed to data analyses and interpretation of the findings. LNA drafted the manuscript. All authors revised the manuscript and had final responsibility for the decision to submit for publication.

**Declaration of Interests**

LA has conducted consulting work for the WHO and the World Bank. He is also the managing director of the global health consultancy Healthier Systems Ltd. NB is a full-time employee and ‘Head of Product and Services’ at Peek Vision Limited. AB is Founder and CEO of the non-profit Peek Vision Group Foundation which wholly owns the subsidiary company Peek Vision Limited. AB is pro-bono CEO for the Foundation and receives a salary from Peek Vision Ltd. All other authors declare no competing interests.

**References**

1. Guest G, Namey E, Chen M. A simple method to assess and report thematic saturation in qualitative research. PloS one. 2020 May 5;15(5):e0232076.
2. Francis JJ, Johnston M, Robertson C, Glidewell L, Entwistle V, Eccles MP, et al. What is an adequate sample size? Operationalising data saturation for theory-based interview studies. Psychology & Health. 2010;25(10):1229–45.
3. Coenen M, Stamm T, Stucki G, Cieza A. Individual interviews and focus groups in patients with rheumatoid arthritis: a comparison of two qualitative methods. Quality of Life Research. 2012;21:359–70.
4. Hagaman A K WA. How many interviews are enough to identify metathemes in multisited and cross-cultural research? Another perspective on Guest, Bunce, and Johnson's (2006) landmark study. Field Methods. 2017;29:23–41.

Appendix 5: Supplementary Table 1: Barrier quotes

| **Themes** | **Quotes** |
| --- | --- |
| **Costs** | |
| Perceived cost of drops/spectacles | - At other service providers like Neema opticians the consultation only is about ksh.1000, so it becomes a challenge. And after checkup, spectacles are about KSH 3,000. (MT40) – tel - I am short sighted and I have problems with light as well. I went to seek for the services but it was too expensive for me. (MT100) – tel - If I could come it could have costed me: in case I was told to buy something like medicine or specs. You know now that could be an issue since there is no money. (MFNG 03) – f2f - When queuing I was told by others who had been attended that there were no medicine, then I decided to leave... After being checked I realized that people were told to go and buy medicine on their own. (MFI01) f2f |
| Loss of wage/income | - I am the breadwinner in my household and Iosing a day is very costly and a hard decision to make. The economy is hard and my family looking up to me. (MT250Z) – telephone - People find it discouraging to go to the outreaches and waste time on long lines. The economy is hard had they have families to feed. (MT010Z) - tel - I am a motorbike rider. I was screened in my village and asked to attend the eye clinic outreach but on reaching the dispensary I meet so many people and very long queues. I was given one of the last appointment numbers in the outreach. I choose to go to work to make money rather than spend my days’ time not knowing whether I will get attended to… If I don’t work, I don’t get money. (MFK008) f2f |
| Transport costs | - Also the fare to come all the way to Meru [from the town I moved to] was a challenge so could not manage to travel on the date given for appointment. (MT09) – tel - I would also have a problem getting to the outreach clinic due to cost, I didn't have fare and that place is far, you have to use almost 150 shillings to and fro, so that is 300 shillings. (MFNG 03) – f2f - Transport was an issue. Travelling from Timau to here its far; around 230 shillings and 300 to go back. My biggest barrier was transport. (MFK2 06) in-person - Transport from Isiolo to Kinoru its a lot so I could not come back. (MFK2 05) – f2f - The main challenge that affected me its transport. I would use around 1500ksh for transport. That is what prevented me from coming. (MFK2 04) – f2f - The distance from [suburb] where I stay to Getembeni dispensary where I was to go for the eye check-up is very far, since I have to use transport of around 70 to 100 shillings to and fro, depending on the day. That was also another challenge why I did not make to go there; because sometimes that might be too expensive for me. (MFI 03) in-person - I did not have fare that day my fare is like 140 ksh. So since I did not have fare I was unable to come. (MFG 06) in-person - "Transport is also a challenge. But you see for me, I did attend to that place, but it did cost me. You see from here home at [location] to Moteteria is 100 bob, so to and fro it's 200 sometimes. It's expensive for some; like for me I used 400 that day on fare, but I overheard another old woman who said that she didn't go because she didn't have transport money. (MFG01) – f2f |
| **Distance and transport** | |
| Distance to the referral site | - I work in Thimagiri market Centre and the clinical outreach was held in Meru town, which is quite a long distance. Even if I had sneaked to attend the outreach, I could not have managed. But If the outreach was held near my market centre I would surely have attended the outreach (MT250Z) – telephone - My home area is Nkobo and on the day of screening I just happened to be a passer-by in that place which I don't know how it's even called. After screening, I was given an appointment for my eye check- up. The place is very far from where I live... So because the place was very far, and I did not know where the check-up clinic was exactly located, I didn't turn up for my first eye check-up. (MT33) -tel - I recently moved to a far located place that was the reason I never attended my appointment (MT28) – telephone - I was screened in Meru but I relocated to [town] after I found a job. (MT984) – tel - I met with the VIP team when I was still staying in Meru town then but later on I relocated to [town] and the distance from there to Gitoru where I was referred for further screening is far. (MT09) – tel - I relocated to home which is [town name] and the distance from there to Gitoru where I was referred for further screening is far - MT09 – tel - There was no station close to me. It was quite far from where I stay (MT 823z) – tel - I would also have a problem getting to the outreach clinic due to cost, I didn't have fare and that place is far... (MFG03) – f2f - Also the distance from Ingoki where I stay to Getembeni dispensary where I was to go for the eye check-up is very far… (MFI03) – f2f |
| **Desire and priority to seek care** | |
| Conflicting work engagement | - I got there around 8 or 9am and found the line was quite long... I could not wait since I was to go to work that day... I could not come back later to the clinic because I work out of town, and I normally come back late at around 8pm (MT40) -tel - I could not manage to attend the clinic outreach since that day I was working. (MT28) – tel - If I was not on duty I would attend (MT028) – tel - On that day I had a meeting… and I thought I wouldn't take a lot of time but I was late to attend the clinic. (MT07) – tel - I went and found that there were a lot of people and it was my work day. I work at a workshop company so I went back. (MT100) - tel - My boss never granted me a sick off to attend the eye clinic outreach because there was no one to cover off my duties. I work in an hotel and there was a big conference that day. I reported to work at 7am and left work at 8pm at night. (MT010Z) - telephone - I did not attend the outreach clinic because I went to Kitale to do farm work, I was instructed by my boss to do the farmwork. I had told him that I had an appointment but he did not give me a chance to attend the clinic (MT174ZZ) - telephone - The day they had sent me for check-up I went for a job I was called to do, then I did not have the chance to go for check-up due to this job. I was sent for clinic at Kinoru and the constructor sent me for work in Isiolo. (MT10) – tel - It was either on Thursday or Friday when I met with VIP people on my way to town when I was checked and referred to Kinoru the next week on Monday, but unfortunately you know I am a hustler, I am a house technician, so I was called same day for some work at Isiolo County… So I had to take the job offered, (MT15) – tel - I was referred on a Monday, which is usually a busy day at work, I got held up at work on the clinic day and I could not be given permission.... If I was not at work that day I would have attended the clinic. (MT17) – tel - I was not around, I had gone for a job in Isiolo (MT44) - tel - I stay in Meru but on the clinic appointment day I had sent my boys to a certain job but they were underperforming and I feared that I might lose the job so I went to Isiolo to handle business. I had thought that the work could have taken around two to three hours but it went on for two days. (MT46) – tel - I did not attend the outreach clinic because I went to Kitale to do farm work, I was instructed by my boss to do the farmwork. I had told him that I had an appointment but he did not give me a chance to attend the clinic… I had to travel to Kitale because that’s where I make my daily bread. (MT174ZZ) - tel - It was on a Friday and the screener told me to go to Igoji dispensary the following Tuesday to get checked up, but that was a very busy day at work I was not given permission from job by my boss. If I got permission I would go to the clinic for the check-up. I tried to ask my boss for permission to go to the clinic and he refused. (MT496) – tel - Yes if I was not on duty I would attend (MT028) - tel - It’s work-related issues - especially on Monday as Monday usually has a lot of work. (MT656) – tel - I was in a far-away place so I was not able to attend the clinic. I had a short and urgent trip'. (MT183z) – tel - I did not get time to go that day, I was at work. (MT504z) – tel - "They had come to my home, since they were going door to door examining people, and they told me that I had problem with my eyes, so they asked me to report to the dispensary by 8am. On that day, I arrived as early as 7:40 am since I wanted to be served first so that I could go back to work. They arrived at around 8am and found that already we were there, though they took time to set their working stations which took time to start, and I think they started at around 9am and I couldn't wait that long ‘cos that was going to mess my job, so I decided to go to work and told myself “if they will come next time and the time was convenient for me, I'd attend”. (MFG 02) – in-person - I was screened in Gathumbee. They were holding a smartphone and asking you to read some letters. My main issue was reading under sunlight and my eyes were itchy. I missed the clinic outreach because I was working in a far place… I was willing to come but held back due to my job (MFNG 01) – in-person - I was at Maua that time, I work for [large corporation] and they can call me in for duties any time in different towns e.g. Mandera and Marsabit. I was planning to come for the eye clinic that day; I do not joke around with my eyes because I know how serious it is. I have been having eye problems ever since I was a small child. Unfortunately during that day I was called to be on duty at 5:00 am at Maua and I had to go. (MFKIA 012) – in-person - I came to the outreach clinic stayed from 9am to 1pm and left, I was called to go for work to mombasa [3:10]. There were many people in the queue, I am a driver, I work in different places, that day I was called and informed that the parcel was ready so I had to go. (MFNG05) – f2f - During the clinic day, the boss said I could not come to the clinic because there was no one at work that day, and it could not be left unattended so I missed the clinic that day. (MFKIA 08) – f2f - I did not come to the clinic that day because I was occupied all day at work that day… I work at a cyber cafe. We open from 8;00 Am to 6;30 Pm but it depends on how the days work is. (MFKIA 06) – f2f - The exercise was also scheduled for the following week but I never managed to turn up because of work. I work at [educational establishment] and since it was not on my off day, I couldn't just fail to turn up for work so that I could come to be checked. (MFK 02) – f2f - That day of the check-up I was at work, and was my work day and if you don't turn up for work you won't be paid for that day (04:19) and even if you ask for a day off, you won't be paid for that day. (MF1 04) – f2f - "I had to go back to my business because I had some clients who were waiting for me, and after doing my work for that day, I did go back to the center again but it was already 5 pm in the evening and there was no one there. (MFG 01) – f2f - In building and construction. You know you can’t just leave work you must wait till the time work is complete… The work is the one made me not to come: I prioritised work. (MFG07) – f2f - [After attending an all-day school meeting] I was to go close the job. (MFK05) – f2f - I mostly drive at night. By the time I get to my destination my eyes are paining, So when the program said, I promised to seek for help. I found a very long line that morning; it’s like people slept there and the clinic had not opened yet. They opened around 8am. There was a queue but you know with many people you can get played. We were given numbers and wait for your number to be called, I did not wait for my number to be called out: I was around number 80. I did not wait. I had a certain job waiting for me, that is why I came early so I can be attended and go back. I asked one of the staff if there is another slot and he told me there was, so I left. I later came back at 2pm and found more people than I left them. (MFK 04) – f2f - When I arrived, I found so many people and I realized ill not be able to get help. I came with my daughter at around 9am. We found very many people. When I arrived at the gate I was told am number 421 and I was hopeful that after clinic I’ll go to work. We stayed till 12pm. I left and went to work. The clinic had about 700 people. (MFI 02) f2f - I came where I was referred at Gakoromone dispensary but there were a lot of people and very long queues. I decided to go back and come back later that day in the evening. I came but still there were a lot of people at 4pm that day. I got other work to do. There were about 100 people. I did not even go past the gate: I came saw the big numbers of people then I decided to go back. I work as a boda boda person. (MFG05) f2f - On that day I was far from home tending to my shamba [plantation]. (MFNG03) f2f |
| Other conflicting engagement | - I was given an appointment but that day I travelled urgently (MTO1) - tel - I was referred at an outreach clinic. That day I was at a school meeting, so I ran late. I did not make it to the clinic on time... I prioritised going to the school meeting since I could be fined if I get late to the school meeting… On the clinic day I decided that I will go again when you guys come back comes back. (MT39) – tel - On that check-up day, I did not manage to attend to that dispensary because I had gone somewhere else. I had gone to a school meeting of my child, and when I came from there it was late. Even though I went to the school meeting, I had in mind that I should go to the eye check-up but I had to attend to the child's school meeting first, and when I came back I did not go to the check-up point. (MT546z) - tel - I was at my neighbour’s event. I thought I could leave by 2pm but my friends were there, and it was an important gathering. I thought I would come back for the outreach but I ended up staying there for two days. I know I made a mistake by not attending the outreach but I have said to myself that if the outreach will be conducted again then I will attend. (MT47) – telephone - I was told to come for the clinic but I didn't make it, I had a meeting in school that same day so i decided to go for the meeting then attend the outreach later. We stayed there all the day. I was hoping I would get time to pass by the outreach but I didn’t make it [2:30].I was willing to attend the outreach but by the time I was coming back, it was already late at 6pm. (MFNG03) – f2f - I went back home two days before clinic day... I did not have to come back from [hometown] since it was far and I did not have any other work engagements in Meru. I prioritised taking care of home chores since no one else was at home. (MFK2 05) -f2f - The day I was referred to come here at Kinoru dispensary happened to be the burial date for my husband’s grandmother. (MFK 03) in-person - My mother was sick, she had a problem with her blood pressure, and normally she stays alone at home and someone had to look after her until she was better... Without that emergency, I was very willing to turn up, because even after coming back, I took my own initiative to seek the service somewhere else - at Meru General . I was checked and I was found to have a problem and I needed to purchase specs costing around 7,000 which I did not manage to purchase because that's too expensive for me. (MFK 01) – f2f - During the clinic day, my leg was so painful I could not come, I was shot by police I later went to hospital and stayed for two weeks (MFKIA 07) – in-person - The clinic day happened to be my child’s schools meeting so I did not make. (MFK 05) – f2f - The screening exercise was much okay, after that I was given an appointment date, which was on a Wednesday, but unfortunately early that day I was called from my daughter's school that she was sick so I had to go and pick her from school and take her to hospital , from hospital, I did come with her to the appointment centre but the queue was long around 30 people already in the queue, it was at around 10 to 11 am, and since I was with a sick child I could not be that patient to wait for my turn to be served. (MFK 02) in-person |
| Forgot | - I also forgot the exact location where I was to go for the eye check-up and no one followed up to remind me of the place and date. (MT 33) – telephone - The message yes came to my phone on Friday but over the weekend I forgot. Its basically forgetting and work priority. (MT656) – telephone - What happened, I lost my phone and was not able to remember the exact date when I was told to show the clinic. (MT36) - tel |
| **Clinical service quality** | |
| Long queue | - I got there around 8 or 9am and found the line was quite long. I could not wait since I was to go to work that day. (MT040) - tel - I went and found that there were a lot of people and it was my work day. I work at a workshop company so I went back. (MT100) – tel - People find it discouraging to go to the outreaches and waste time on long lines. The economy is hard had they have families to feed. (MT010Z) – tel - I went where I was referred at the chiefs camp clinic and found out that there were a lot of people... Then I was puzzled how I will be attended… Then I decided I’ll go some other date. (MT34) – tel - There were very many people in the outreach clinic. Most of the people in the queues were not attended and the place was so congested, there was only one dispensary but people were so many. I had someplace to go by 10am so I left because I could not wait any longer (MT48) - telephone - I went in the morning at the dispensary which I was referred to but there were way too many people. I had other personal errands to run. I did not want to waste time there because the line was moving very slowly. [I came back at] 5pm and the people were still crowded at the facility… I figured the poor service was because the clinic was free. (MT147) – tel - It got to 6pm and I went back home… I had not be attended since morning: I went from morning until evening and I had an eye problem but I had to endure. I completely left that process (MT533) - telephone - When I arrived at the outreach clinic, the turnout was very huge with long queues. I was very uncomfortable. I dislike long ques because I am an introvert. I arrived at 9am and stayed up to 1pm then I left. I felt like I was wasting my time. If I waited I would be attended to at the evening. (MT772) - telephone - People were too many. I was number seven-hundred-and-something. There were around more than 1,000 people. I came next week Monday but I was not attended as the people were very many (MT 952Z) – tel - I overheard that day that the line was long that day. You know someone can get tired of the line, maybe you have work to do later… I went later only to find the long line: I thought I could go and get attended quickly [but] I had to go back to work (MT504z) – tel - I came to the outreach clinic stayed from 9am to 1pm and left. I was called to go for work to Mombasa [3:10]. There were many people in the queue, I am a driver, I work in different places, that day I was called and informed that the parcel was ready so I immediately left. (MFNG05) – f2f - I attended the clinical appointment at Kianjuri but I met a lot of people and the queue was so long. I stayed for around four hours, till 1pm, I lost hope of being attended to and I went home… I also met a lot of elderly people who were more in need of the eye services than me so I thought It was better for me to give them a chance to get treatment and I could come another day or time. (MFKIA011) – f2f - I had the option to go to the appointment on either Monday or Friday. I selected Friday because I'm busy working on Mondays. However, when I arrived at the outreach on Friday, there was a large crowd, and large crowds, and I didn't receive an appointment number. When I inquired, I was informed that all appointment numbers for the day had already been given. I was not given an appointment number and I was not attended to. People prefer to go to private hospitals rather than wasting a lot of time in the queues. (MFK009) - f2f - I am a motorbike rider. I was screened in my village and asked to attend the eye clinic outreach but on reaching the dispensary I meet so many people and very long queues. I was given one of the last appointment numbers in the outreach. I choose to go to work to make money rather than spend my days’ time not knowing whether I will get attended to. (MFK008) – f2f - I also asked around and was told there was a very long queue. (MFK05) – f2f - I mostly drive at night. By the time I get to my destination my eyes are paining, So when the program said, I promised to seek for help. I found a very long line that morning; it’s like people slept there and the clinic had not opened yet. They opened around 8am. There was a queue but you know with many people you can get played. We were given numbers and wait for your number to be called, I did not wait for my number to be called out: I was around number 80. I did not wait. I had a certain job waiting for me, that is why I came early so I can be attended and go back. I asked one of the staff if there is another slot and he told me there was, so I left. I later came back at 2pm and found more people than I left them. (MFK 04) – f2f - When I came I found a very long queue. The line being long made me to leave… There were about 600 people. There were also some people who did not follow the order of the queue using the provided numbers, some people. (MFK01) f2f - I realized I’ll get late for work, so I went back. Then at around 10am I came back and met a very long queue. (MFI 01) f2f - When I arrived I found so many people and I realized ill not be able to get help. I came with my daughter at around 9am. We found very many people. When I arrived at the gate I was told am number 421 and I was hopeful that after clinic I’ll go to work. We stayed till 12pm. I left and went to work. The clinic had about 700 people. (MFI 02) f2f - On that day, I did not have much time on my side, but I did come, but it was already late at around 11 in the morning, and other people had already come and there was a long queue, and there was no way I could have maybe been excused to receive the services then go back to my work. (MFG01) f2f - I came where I was referred at Gakoromone dispensary but there were a lot o people and very long queues. I decided to go back and come back later that day in the evening. I came but still there were a lot of people at 4pm that day. There were about 100 people. I did not even go past the gate: I came saw the big numbers of people then I decided to go back. I work as a boda boda person. (MFG05) f2f |
| Insufficient numbers of staff | - The line was moving very slowly because there were not enough eye doctors attending that day(min 5:30)I have tried looking for help in several facilities like the general hospital but the medicine does not help usually so i was looking for something different that day, only to be delayed the whole day. I figured the poor service was because the clinic was free(min8) (MT147) – telephone - I came next week Monday. I was not attended as well people were very many. There were very few doctors attending the large crowd: there were about three doctors. (MT952z) – tel - I felt like the doctors were rushing with the treatment so that they may cover many people. (MFK 04) – in-person - The clinic had about 700 people. Doctors were 3-4, and using one room. (MFI02) f2f |
| **Facilities** | |
| Clinic not open at stated times | - On 21st I arrived at around at around 3:30pm but I found that they had already closed their work for the day. We met with the doctors at the door and they told me that they were not taking any more patients at the time. There were still many people who did not get attended that day (MFNG 03) – in-person - The day I was referred I came at 8:00 am and met the gate was closed. There were no people and because I was at work I realized I’ll get late for work so I went back. (MFI 01) – f2f - Doctors also came a bit late; around 10am. (MFI02) – f2f - They asked me to report to the dispensary by 8 am. On that day, I arrived as early as 7:40 am since I wanted to be served first so that I could go back to work. They arrived at around 8am and found that already we were there, though they took time to set their working stations which took time to start, and I think they started at around 9am. And I couldn't wait that long coz that was going to mess my job, so I decided to go to work and told myself if they will come next time and the time was convenient for me, I'd attend. (MFG 02) f2f |
| **Awareness & communication** | |
| Lack of clear information on services available | - They did not tell us if we need to come with money or not. Eye treatment, we are usually told to come with money. I assumed I’ll go there and they will ask me for money and I did not have it. MFK2 04 – in-person - The most important barrier is when I realized that even if I come ill not receive help. She told me even if you go you will not receive help you will be sent to general hospital so you will waste the whole day and there will be no help. (MFG 06) – in-person |
| Lack of clear information on appointment days | - The following day, I did not go to the centre because I knew that those who have specialized with eyes wouldn't be there, something I heard from my neighbours. (MT546z) - tel - We were not told how many days the clinics would go for, I was only given a single day. I did not know if it was running for other days. If I knew the days I would come, you know here I don’t have to use fare, it’s just a walking distance (MFK 03) -in-person |
| Lack of clear information on appointment times | - I was not sure when they open and close. (MFKIA 06) – f2f - I did not know about the opening and closing times. I thought it’s like the normal working hours; form 8am to 4pm (MFK O5) – in-person |
| Insufficient counselling | - They just told me that I have problems with my eyes and I should visit Ingoki dispensary so I did not know what I was going to do there, is it surgery, is it being given medication, is it being tested again? And for me I have always known that my eyes are okay, and on that day they told me that they are not okay. They were very brief and I didn't know what to expect, so that shock of being told that I have an eye problem which I have never had before is the reason why I did not go. (MFI 03) – in-person |
| Did not receive reminder message | - … and no one followed up to remind me of the place and date of the check-up since I never received a message to remind me of the scheduled appointment. (MT 33) – telephone - You know the date came and they had told us the would call and they never called… I did not get any [SMS] message. (MT823z) - tel |
| **Fear** | |
| Fear | - "Another thing that hindered me from reaching that place of check-up was because of fear, you see this place is not home, and I don't know people here you can tell someone to take you to some place and they might end up raping you or taking your property so you so you will probably be filled with fear. Another reason of fear, it's because it involves the eyes many people are fearing since that incident of Worldcoin so it's not easy to trust anyone. And you get in a group, you're the only one who agreed to be screened so the others will discourage you from going, telling you that you'll be killed or something bad will happen to you, because of the recent activities of so many online scamming, it's not easy to go to receive help even when you have a problem because you can't trust anyone. (MF1 04) in-person - Then I was scared because of the Worldcoin thing so I developed some fear. This also made me not to come for clinic that day. (MFK2 05) – in-person - My eyes have been having problems since I was young but I have never sought for treatment, despite foreign actors always coming to give us free service. My mother used to tell me that you do not joke with eyes, you don’t give everyone access to your eyes because some may be bad people who may put bad substances on your eyes because her brother once did that and his eyes got worse with time. That’s why I feared to seek treatment. (MFK 03) in-person |
| **Norms, values, health beliefs** | |
| Discomfort with mixed genders in the queue | - There were women on the line. They could have different lines for youths and women for some us to be comfortable because it is shameful to be on the same line with women and children, with worries how they will perceive me as young man. It was a challenge for me to just stand there with women… I had to go back that day without being attended even though right now my eyes have a problem. (MT040) - telephone |
| Male health seeking behaviour | - As a man it is very hard to prioritize my health as I am manly focused on my family’s wellbeing and It is easy to forget my health needs (MT250Z) - tel |
| Assumption that eye problem wouldn't be addressed | - I am short sighted and I have problems with light as well... I was told that they were only treating eyes defects like short sited and long sited people and they were not giving glasses for protecting light. (MT100) - tel |
| **Other** | |
| Dislike crowded places | - When I arrived at the outreach clinic, the turnout was very huge with long queues. I was very uncomfortable. I dislike long ques because I am an introvert. I arrived at 9am and stayed up to 1pm then I left. (MT772) - telephone |
| Sought services elsewhere | - After the screening I was told to go for the referral clinic at Meru Prisons. That day my eyes were red and had discharge. They gave me a later clinic date. The condition of my eyes was worsening daily and I could not wait for the clinic. Light exposure made my eyes red and they would start tearing so I had to look for an alternative. I went to Meru general hospital, I was given medication and my eyes are now so okay. (MFK 02) - in-person |

Appendix 6: Supplementary Table 2: Solution quotes

| **Theme** | **Quotes** |
| --- | --- |
| BARRIERS | |
| **Costs** | |
| Provide transport fare | If I can be provided with fare, I would definitely attend the clinic because the challenge is income and moving to Gitoru requires money - MT 09 – telephone  According to how the economy is, if we can be given fare, at least you can borrow some money for transport to attend the outreach, then refund later. You can also help people after screening at least give some assistance, you can tell us to look for some money and then you top up so that we can manage to buy medicine and specs since if its only coming to be checked, even if I keep coming again and again it will not be of much help. MFNG 03 –  I also feel like, at least we should be provided with fare - MFG 01 – |
| Subsidise treatment | You should consider needy people who cannot afford [spectacles]… At least organise yourselves and look for the needy to help them, If I get that money I can go and get the spectacles because no one is happy when their bodies have a problem. I cannot even read the bible or the numbers on the phone - MT 148 – tel  According to how the economy is, if we can be given fare, at least you can borrow some money for transport to attend the outreach, then refund later. You can also help people after screening at least give some assistance, you can tell us to look for some money and then you top up so that we can manage to buy medicine and specs since if its only coming to be checked, even if I keep coming again and again it will not be of much help. MFNG 03 – |
| Pay people to attend | You should also give people some little money if they attend the appointment - MT 46 - tel  I'm asking if we can be compensated with the amount we could have been paid because we leave our job so at least you are sure - even if you're not going to work on that day - your children are not going to sleep hungry. Because some of us, we're the providers of our families. MFI 04 – |
| **Distance and transport** | |
| Move clinics closer to where people live and work | The outreach should be situated in a place which is easily accessible to many people and where many people live... It should not be very far from where people live - MT 28 – tel  Because I was just a passerby, it will be better if the eye screening was brought to where I live in Nkobo – MT33 – tel  Have the stations close to the people where we can all reach. MT 823z – tel  I can go to the outreach clinic if its situated near me where i will get help but not going all the way to Meru MT682 - tel  If the outreach was held near my market centre I would surely have attended the outreach – MT 250Z – tel  You should bring the services closer to the people… Because you have the people’s contacts and where they live, you can conduct an outreach in a place which near to where they live. MT 183z - tel  They should bring the clinics near the people to cut cost on transport - MFK2 04 –  Services should also be near - some facilities are at a long distance so it becomes difficult to go there. - MFK 009 – in-person  For distance, I think you should organize and bring the services to someplace that's near to us for example chief's camp - MFI 03 – in-person  Clinics should be set in various places in every county. So that the clinics would be near people. MFK2 05 – in-person  I am suggesting if they could take the services to every dispensary and for my case, a near dispensary like for me, [name] dispensary. - MFG 01- in-person  Also, it would be easier if they could bring the services to nearer facilities for instance for people from my area, to [name] dispensary - MFG 02 – in-person |
| Provide transport | If you give me transport and day to come, I will come. MT 10 – telephone  For the elderly you can look for means of transport, many of them will come. You can organise with people like chiefs so that they can know how to take people to the places where the services are offered. - MFK 009 – in-person  if they would be able to provide transport that would be OK. MFG O7 – in-person |
| Door-to-door services | Is it possible to have a doctor visiting them [elderly people] door to door? It would be better for those who aren’t able to go there - MT 15 – tel  If possible, you can take the services to people's homes considering there are those very old who cannot make it to the appointment centres - MFK 01 – in-person  Consider door to door services but I don't know if that's possible. MFI 03 – |
| **Clinical service quality** | |
| Improve punctuality and speed of staff | I think those that are providing the services should be punctual and fast - MT 07 – tel  Increase [the number of] doctors from four to ten, and then those doctors to arrive early at around 8am– MFI 02 - in-person  The solution is still time, though 8am is still okay, but I think if maybe they could work on their organization so that much time is not wasted on preparing the working stations - MFG 02 – in-person |
| Ensure that all medicines and glasses can be provided on-site | Equip more drugs in the dispensary - MT 48 – tel  When you see someone, make sure they get medication or give them spectacles; not writing prescriptions and referring them to chemists. I think this does not help. You should have all the medication for all allergies and spectacles, not referring people to places - MT 533 – tel  After being screened you should receive all the services like medicine and everything, you get solution for everything but not again looking for services from other places. MFNG05 – in-person  Also, the program should improve on their equipment and machines used for examination, so that the services will be satisfactory. MFG 01 – in-person  Next time let the doctors be many and let them come with medicines. MFI01 – in-person  For the issue of specs , maybe you can add more resources that are enough for everyone next time. MFG03 – in-person |
| Add more staff at each clinic | Add more eye doctors that day so that they may help each other in covering more people on the clinic days - MT 147 – tel  Have many check-up doctors, not bringing two or three of them. That day they were only five or six, you see them attending all those people - is impossible - MT 533 – tel  Increase the number of doctors so that the clients can be served more efficiently and on time - MT 772 – tel  Add more doctors so that everybody will be served - MT 952Z - tel  Maybe you should add more doctors to attend to more people so the lines can move faster - MT 504 Z – tel  Those people doing screening should be many because staying for two three hours with this economy is a loss. MFNG05 – in-person  There is a high number of people requiring eye services thus the number of doctors should increase - MFKIA 011 – in-person  Increase the staff because there were very many people increase the doctors from four to ten - MFK2 04 – in-person  Increase doctors from four to ten. MFI 02 – in-person  Increase no of doctors so that I will not take long waiting because I have other duties to do. Next time let the doctors be many and let them come with medicine… Add doctors from three to six: that would be OK. – MFI 01 – in-person  The doctors attending people should be many. Because like… people were so many, and most of them went back without being attended to. So if there were many doctors it would have been easy. MFK 009 – in-person  I think next time you should increase the number of doctors attending so that every individual may get good quality service - MFK 05 – in-person  Maybe next time you can add more manpower because for the long queues and stuff… I have a feeling that there are those who never got attended to at all because of lack of enough time, due to inadequate staff - MFG 03 – in-person  …and set more stations, and also doctors to be added in those stations. MFG 05 – in-person |
| Schedule fewer people to attend each day | When you register, register few people… Having 1000 people wastes a lot of time and you cannot handle such a crowd. If you target 100 people, you will attend all of them. Having many people will have them trolling around all day. You see, I had an eye problem but I swore never to come back again, I would rather go to the hospital than come waste time around just because it is free - MT 533 – tel  The outreach should limit the number of clients they are attending to. It make no sense to refer 600 people and they cannot fully attend to in a day. They should choose a much smaller number comprised of people they had referred. MT 772 – tel  You should take time to examine every patient as it should without minding the workload ahead, that’s why I think you should schedule less people. MFK04 – in-person  I would also like it if you control the number of people you schedule for a particular day, because now you have experience of how many people you can attend for a day. Schedule just that amount of people so that everyone may be attended to… It wasn’t fair of you to call that many people, they were already sick and they had to wait for hours under the scorching sun. MFK01 – in-person |
| **Facilities** | |
| Keep the clinics open for a greater number of days | The screening exercise should not be done just once, it should be repeated maybe even for three days during weekdays before an appointment for the first check-up is made - MT 33 – tel  Maybe you can add more days so that you can schedule few people on one day - MT 147 – tel  You should extended the number outreach days to attend to more people - MT 001 – tel  If there were more days I could have attended the clinic. I suggest that you add more clinic days - MT 39 - tel  Come at least two days consecutively; let it not be one day. If I miss one day, I will be able to go the following day - MT 34 – tel  Add more days for the clinic so that people who did not manage to attend the clinic on their appointment dates can still get a chance to be treated. Many people have eye problems but they are also busy looking for ways of surviving during these tough economic times - MT 174Z – tel  You should extended the number outreach days to attend to more people - MT 01 – tel  Also you should add the number of outreach days to attend to more people. MT47 - tel  Add more clinic days around 3-4 days - MT 952Z – tel  For me I'd suggest that next time you have a similar exercise, at least add more days because this time you had only specified for one day, this will assist those who hadn't managed to reach that day to have another chance. MT 546z - tel  Add more days. You see I was referred for one day if there was a next day I would purpose to come - MT 504 Z – tel  Having a single day for clinics gets people congested… I think that Monday, Wednesday, or even the whole week of clinic will be easier for most people to access the clinic as it is easier to ask for permission from work and be sure you will be attended. With the whole week one can get a day to go and if they do not get attended that day, they can still choose the next day and will be sure to be attended. MT040 – tel  You should also do those outreach clinics for more days at least two or three days in a month. That time they were doing the outreach for just one day. MFNG05 – in-person  The outreach should not be in one day. It should be at least two days in a week. If you allow everyone to the outreach there will be long queues and many of them will not be attended. MFKIA 012 – in-person  You can also add days, if I miss today, I can come the next day. If you add the days, and make sure you remind us, it is okay. MFKIA 08 – in-person  If you add the clinic days I feel it is okay. MFKIA 07 – in-person  You can just extend the clinic days to have more of them, like four to five days. MFKIA 06 – in-person  More appointment days maybe for like 2 weeks. MFK 01 – in-person  If possible you can also add some days like then you were checking people on Monday and Friday because many people were interested but did not get help. MFK 009 – in-person  If it is possible, have the clinics for at least two days: if you miss today, you can purpose to come the following day because even those people were too many. MFK 05 – in-person  You know if you give the clinic like three days, you can even travel for a safari, come back the next day and still attend the clinic. So I would suggest you schedule more days for the clinics. MFK 03 – in-person  Look for a day like Friday because work load is few. MFK04 – in-person |
| Extend clinic opening hours | If they would extend services to the evening at 5pm that would be okey since most people are out of work. MT100 – tel  I would say that you guys had a great job in our community according to many testimonies from the people who made it to the clinic and I regret not going, I feel devastated. However I would suggest that you add some more opening and closing hours so that people who are committed during the day can access the clinic. MT39 – tel  Open the clinics early and have the two days and people coming early. MFK 05 – in-person  Also have the clinics start as early, like if we come around 6:30am the clinics can open at 7am. I feel like that days clinic started late they started at 8am, I was there at 6:30am. MFK04 – in-person  Also if it's on weekdays, if it's possible if the exercise could extend the working hours, let's say from 4 pm to 6 pm, that one too I could manage, because I would have come from work. MFK02 – in-person |
| Keep the clinics open on the weekend | The biggest challenge for me was timing. If timing was ok I would have attended; like weekends I'm not usually very busy. MT100 tel  You should have clinics on Saturdays because that is when most people are not held up at work. Having the clinics on Monday is hectic as people are held up at work and getting permission is quite difficult... Sometimes bosses do not grant day offs on busy days, especially week days. If you could make the clinics to be on a weekend many people will attend, I will also attend if you make the clinic day to be on a weekend. When you make it on a Saturday many people will attend because even if they are busy they get off from work quite early at around 1pm. MT17 – tel  I am always free on Saturday and Sunday… I think that Monday, Wednesday, or even the whole week of clinic will be easier for most people to access the clinic as it is easier to ask for permission from work and be sure you will be attended. With the whole week one can get a day to go and if they do not get attended that day they can still chose the next day and will be sure to be attended. MK040 – tel  Maybe if the exercise could be scheduled off working days - that's during the weekend, in Saturday or Sunday, because at that time am free without work I could turn up. MFK02 – in-person |
| Add more clinic locations | Have more many work station where people can easily access the services to reduce the distance people need to travel to get treatment. Even the elder people can reach the outreaches also it give them option on where they can get treatment because on line on a particular station but be short where as the other might be long. MT 010Z – tel  Some people should be referred to other hospitals like MTRH. MT48 – tel  Have eye medics even in Nyeri. MT984 – tel  Next time there such a project, its best if you people divided the community people according to their villages or maybe sub-location 9:10 so that people don't overcrowd in the same place like that day because I think that day the whole location was referred to Kambiti which made the area overcrowded and we could not receive the services because the people were many. MT38 – tel  Set more clinic centers so that people will not need to travel from far. MFK2 06 – in-person  You should have many stations and also the doctors attending people should be many. Some other places can be used like churches and it will be easier for people to go to access the place. MFK009 – in-person  You know so many people have eye problems like all those that were that day. Next time have many centers, like three, not one like that day. MFK04 – in-person  Let there be more facilities for the clinics, like five stations. That would be OK. MFG07 – in-person  If there will be more stations for check-up that would ease the long queues. Even the clinics should be set up even in schools nearby, then people would not be as many that day. At least five stations would be good in this area of Gakoromone. Come with machines even to the nearby schools and set more stations, and also doctors to be added in those stations. MFG05 – in-person  For the long queues, am suggesting if there will be more screening centers to avoid overcrowding at one center and the long queues. MFG03 – in-person |
| Mop- up clinics | The outreach should be conducted another time so that the people who were left unattended could be attended. MT48 - tel  If the outreach is done once more I could go. You should come back because there more people who were not attended to - MT47 – tel  Just tell us when you are coming back to the community for other clinics. Even this week you can tell us you are coming and we shall attend… Plan for when you are coming next, three days this time will be better so that if someone misses one they can still have a chance. MT496 – tel  Let's say, those people who never managed to attend on that particular appointment day, they should be followed up immediately maybe later that day or the following day so that their appointment can be rescheduled. For me, I would have wished that the exercise would be repeated for more days following up on those who missed the opportunity. MFK01 – in-person |
| Give each person a specific appointment slot | The attendance number in the outreach should be issued in line with the referral messages clients received. The message should also have the attendance number and the time allocation to help the clients organize themselves to attend. MT772 - tel |
| Schedule fewer people to attend each day | I think you should schedule less people. MFK04 – in-person  I would also like it if you control the number of people you schedule for a particular day, because now you have experience of how many people you can attend for a day, schedule just that amount of people so that everyone may be attended to. That way everyone will be aware what time they will attend so that they may not waste the whole day. It wasn’t fair of you to call that many people, they were already sick and they had to wait for hours under the scorching sun. MFK 01 – in-person |
| **Awareness & Communications** | |
| SMS reminders, including on the day of the clinic | Sending them SMS message and remining them to attend their clinic appointment or calling them. MFK008 – in-person  That same same day for the clinic I should receive a reminder message to remind me to attend. I have many things I can easily forget… A reminder message should be sent before and during that day to attend the clinic. MT656 – tel  They should make announcements early; not a day before the clinic.. They can make announcements three days before so that you can be prepared. Even sending messages four days before, its OK. MFK2 05 – in-person  Next you send me a message to remind of the clinic and a phone call will be okay to remind me of the appointment. MT36 - tel |
| Send a text to individuals who missed the outreach clinic to invite them to attend on another day | If a person never made it to the outreach, he should be notified of the next eye clinic outreach even if it is in another location. Because one cannot be busy all the time. MT46 - tel |
| Phone call reminder, especially for those who cannot read | Next you send me a message to remind of the clinic and a phone call will be okay to remind me of the appointment. MT36 – tel  Sending them SMS message and remining them to attend their clinic appointment or calling them. MFK008 – in-person  If someone didn’t make to attend the outreach, you can call the to remind them. MFNG03 – in-person  Maybe you can remind us by calling us as you have today. MFKI08 – in-person  In order to create the trust and be sure that this exercise is medical, at least you should call on the day of the check-up so that you can explain the exercise in detail - so that we can get to trust the exercise because sending just a message as a reminder is not convincing enough or satisfactory. MFI 04 – in-person |
| Explain why attending clinic is important at the point of referral | Also during screening we should be given more information why this clinic is important. MT656 – tel  They should also be informed the importance because some people may end up blind. MFNG03 – in-person |
| Use churches and radio broadcasts to remind people to attend | Mobilize the clinical outreach especially on churches and radios. MT48 – tel  Also, the activity should be announced and advertised to people in public, for example in TV or radio shows. At least even if I don't know you, someone else might have seen you, so people will be able to come more freely without the fear of being scammed or conned. MFI 04 – in-person  It should be announced in public so that everybody will get there because the message can come when the phone is off. Let it be publicly announced that certain day will be clinic day, we meet at a specific place. MFG 07 – in-person |
| Specify clinic dates and times | I would propose that if you have a project like that one you communicate well on the exact opening and closing times, so that people may not come and get stranded outside and eventually go home without getting treated. There were so many people outside the facility who got devastated because they thought that the exercise would go on at least up to close of business hours. I feel like if you communicated well on the closing hours I would have programmed myself well and come in the morning whereby I could get the treatment that I required. MFNG03 – in-person  You should communicate on the time because I did not know that the clinic was opening early, and tell the people to come early enough so that they may be attended effectively. MFK04 – in-person  Communicate well on how many days you will be available, when I know the duration which you are staying I will avail myself. MFK03 – in-person |
| Allow people to choose their appointment day | Next time they should ask me when I am available instead of putting a date for me because of my work. The biggest challenge for me was timing. MT100 – tel  Communicate with client if they will be available for the outreach. If not allocate them another day and time. MT894 – tel |
| Clarify what services are available at the outreach clinic and specify if there are any costs linked to these services | Clarify information because of the Worldcoin thing, so during screening we should be given more information. Anything free people think its hidden things. MFK2 06 – in-person  People should also be announced that they will be helped, not only will they be screened, there will be other help. MFNG03 – in-person  They should tell me that the services are free so that ill not assume that there are charges... they should tell us the clinics are free. MFK2 04 – in-person |
| **Norms, values & health beliefs** | |
| Sperate younger people, women & children, and elderly people | Address the line issue and have women on their [own] lines. But youth can just have their line where we can mingle and interact - for example on how hard the economy is - and by the time we realise the line is shortening. But with women on the same line we will not have anything to talk about. MT040 – tel  One day of the outreach should be reserved for the elderly and the other day for the other people since you should give priority to elderly people. If you allow everyone to the outreach there will be long queues and many of them will not be attended. MFKIA 012 – in-person  What I would want you to improve is that next time hire competent queue managers who control how people are coming in, and the elderly, pregnant mothers and mothers with small children to be put on their own queue so that there will be effective treatment. MFK01 – in-person |

Appendix 7: Original drafts: Intervention counselling script and SMS reminder

**Original enhanced counselling script**

I have found a problem with your eyes. I am referring you to the outreach treatment clinic that will be held at [location] on [date] between [time] and [time]. At the clinic, eye care professionals will perform a specialist assessment and provide medicines and spectacles as required, all free of charge. Note that a small proportion of people who attend the clinic will be found to have complex eye problems that require onward referral for hospital assessment and specialist glasses. This may incur a cost. However, the vast majority of people have their needs fully met for free at the outreach triage clinic and do not require any further referral.

With treatment, you will be able to see more clearly. This will help with your work, reading, viewing screens, and many other things. It is important that you attend the clinic or your eye problem may get worse. The clinic will only be running from [day] to [day], so if you don’t manage to attend, you may not be able to get free care again in the future.”

**Original intervention SMS script**

We found that you had an eye problem. Please attend the outreach clinic at <<location>> on <<date>> between 9am-5pm to receive free medicines/spectacles

If you are found to have a complex eye problem, you may be referred to a hospital for further care or specialist glasses, and this may include a fee

However, the vast majority of people who attend the outreach get their eye problem fixed, for free, without the need for any further referral

It's important that you attend, as your eye problem may get worse, and you might not have a future opportunity to access free care. See you on <<day>

**Changes**

We made three changes based on feedback:

1) One of the young men from the left behind group wanted us to change the sentence *"It is important that you attend the clinic or your eye problem may get worse"*, to make it ‘less scary’. We had included this original sentence based on interview feedback that we needed to better explain the importance of attending. For instance: “They should also be informed the importance because some people may end up blind”. We changed the wording to read; “It's important that you attend to protect your vision.”
2) Due to inflation-imposed budget constraints, the clinics are no longer able to provide all glasses and medications for free. At this point in time it is not clear how subsidised the costs will be. We removed wording stating that the treatments will be free. We kept wording stating that the assessment will be free as this still represents a major advantage over attending a high-street provider or hospital clinic.
3) Representatives from Peek vision advised that “see you on <<day>>” might be misleading, as the day of the appointment might be more than a week away (e.g. next Friday rather than this Friday). We changed this auto-populated section to “see you on <<date>>”.

Appendix 8: Barrier and solution quotes from the interviews with high-income people

| **Themes** | **Quotes** |
| --- | --- |
| BARRIERS | |
| **Costs** | |
| Perceived cost of drops/spectacles/services | - There is a friend of mine who was also referred to general hospital to buy specs which were costing 3500 shillings [7.37]. (HIG7) - I just know if I come for the clinic, I will be told I have a problem and I will be required to pay for the services but if I know I will get free services I will be motivated. (HIG4) |
| Loss of wage/income | - N/A |
| Transport cost. | - The day of appointment I got a job transfer from Meru to Maua (2:50). It’s the transport from Maua to Meru the fare is too high (HIG3) |
| **Distance and transport** |  |
| Distance to the referral site | - N//A |
| **Desire and priority to seek care** |  |
| Conflicting work engagement | - I was given a date to be checked but that day I went to work in Nairobi, we had other programmes in Nairobi that day on insurance that’s why I did not make it [2.35], I work with insurance and I often go to Nairobi for work, I don’t have specific time or date to go to Nairobi but mostly I usually go on Thursdays and Mondays [4.30]. (HIG1) - I didn't attend the clinic since I travelled to Isiolo and that day I was working in Isiolo, [3.10]. It was a working day, and I was on duty, I didn't get some time to attend the outreach clinic. (HIG2) - The day I was supposed to go for clinic i went back at work in Nyeri. The day of clinic appointment was the same day i went back for work. (02:42) (HIG5) - There is a place I went that day, I went to work in Naivasha, I don’t work specifically in Meru, the work I do is usually anywhere like now I am in Maua [1.05], I do fitting, I fix things like gypsum and wardrobes. I can come another day especially from next week because now I am free [2.25]. (HIG6) |
| Other conflicting engagement | - I was screened and told that my left eye had a problem, was referred to Kinoru dispensary but I was sick that day. That whole period I was unwell and that specific day I was weaker (HIG4) - I was told to first buy a certain drug and then go to general hospital in case I don't improve [1.15], after using that drug the problem cleared so I dint go to the general hospital. I had told them I wouldn’t get time to go to the hospital they told me to buy that drug I use before I organise myself [4.00]. I used that drug for just one month but the problem cleared totally (HIG7 |
| Forgot | - I was screened and told that my left eye had a problem, was referred to Kinoru dispensary but I was sick that day and I also forgot about the clinic [1.43]. (HIG4) |
| **Clinical service quality** |  |
| Long queue | - I went and found that the queues were very long at Kinoru dispensary (2:23) I went around 10am.i stayed for about an hour. And I work at Makutano, so I went back. (HIG3) - The queue was too long, people were so many, so I just went back to job [0.52], where they were offering the service was different from where we see patients, we were not sharing the clinics we had given them a separate place but within the same compound [1.35]. (HIG8) - Reason why I did not go is that I used my card and I decided to go another hospital and use my card instead of Meru general hospital, where I was referred. I went to life care hospital. Even if I go to general ill find long queues (11:30) (HIG10) - I went around 3 pm and the queues were very long around 2000 people many people did not go to see doctor due to long queues still at 3pm there were a lot of people (4:34) then i decided to go back home (HIG11) - I was worried about going to the hospital because there is usually so much queue (HIG7) |
| Insufficient numbers of staff | - N/A |
| **Facilities** |  |
| Clinic not open at stated times | N/A |
| **Awareness & communication** |  |
| Lack of clear information on services available | - N/A |
| Lack of clear information on appointment days | - We were not told the specific day to go to the hospital, maybe we can be told to go a specific day and be given a note so that one can know that a certain day he/she will be going to the hospital. (HIG7) |
| Lack of clear information on appointment times | - N/A |
| Insufficient counselling | - N/A |
| Did not receive reminder message | - N/A |
| **Fear** |  |
| Fear | - N/A |
| **Norms, values, health beliefs** |  |
| Discomfort with mixed genders in the queue | - N/A |
| Male health seeking behaviour | - N/A |
| Assumption that eye problem wouldn't be addressed | - N/A |
| Dislike crowded places | - N/A |
| Sought services elsewhere | - N/A |

| SOLUTIONS | |
| --- | --- |
| **Costs** | |
| Provide transport fare | - Provide us with transport, if provided with fare I can come, 730 shillings will be okay for me [15.12], I can request for permission to go to the clinic for few minutes because people get permission even to go to hospital when they fall sick, [15.39] (HIG2) |
| Subsidise treatment | - If you could have convinced me and promise me that I would get spectacles for free, I could get motivated [8.15]. I just know if I come for the clinic, I will be told I have a problem and I will be required to pay for the services but if I know I will get free services I will be motivated. (HIG4) - I wouldn’t even forget to attend the clinic [8.24], for example if you offer things like operation for free or even lower the cost of the services then people would be so motivated (HIG4) - That drug was so costly, I bought it at 1050 at [location] chemist [4.17], many people would not afford to buy drugs so in future you can have variety of drugs and even different spectacles even if we can pay for some amount, you get served once [7.50]. There is a friend of mine who was also referred to general hospital to buy specs which were costing 3500 shillings [7.37]. (HIG7) |
| Pay people to attend | - N/A |
| **Distance and transport** | |
| Move clinics closer to where people live and work | - Since you people are concerned and are following up on us, you can even come to the remote areas for those who can’t access the clinics in town since there are so many people with eye problems, like the way CHVs works, you can even take people like me to go round to remote areas since there are mothers who can’t reach the clinics in towns. you can also get to other places like Samburu rather than proving services only to Meru County, it was even in media that a place like Samburu people have eye problems so you can visit such places as you observe what people are going through, [9.12] (HIG2) - If I can do my appointment where I am e.g. Nyeri or Kirinyaga, if the service could be brought near my working area (04:44) (HIG5) - Bring services closer to people especially in rural places (4:48) in a school or a public ground (HIG9) |
| Provide transport | - N/A |
| Avoid setting up clinics in areas with difficult terrain | - You should also avoid rural areas where roads are not accessible e.g. that time it was raining and therefore most of the people wouldn’t make it to the clinic, [7.40]. (HIG1) |
| Door-to-door services | - N/A |
| **Clinical service quality** | |
| Improve punctuality and speed of staff | - N/A |
| Ensure that all medicines and glasses can be provided on-site | - N/A |
| Provide high quality spectacles/frames | - Next time come with spectacles of good standard, not for the old people. Come with glasses with good standard (6:39) (HIG3) - ...and next time come with spectacles of different types and medication (HIG10) |
| Add more staff at each clinic | - Adding the number of medical staffs so that people can be attended fast, if I had gone to the clinic and found a long queue I would just go back since I can’t keep waiting in the queue, If someone come from far and the services are given fast, he/she can’t feel like they are wasting time. (HIG1) |
| Schedule fewer people to attend each day | - if they do it again, they say a particular area should be attended at a particular day this will avoid overcrowding and long queues... (HIG10) |
| **Facilities** | |
| Keep the clinics open for a greater number of days | - Adding the number of days to be twice a week or if its monthly once a week (HIG8) |
| Extend clinic opening hours | - N/A |
| Keep the clinics open on the weekend | - If it was on weekend Saturday I would attend (03:21) (HIG5) - you should do the clinics during weekends especially Saturdays [4.34], a day like Wednesday is very tricky, most people work the whole week then get paid on Saturdays so they can make it to attend the clinic [5.24]. (HIG6) |
| Additional workstations | - many stations to see people and to have many doctors (5;15) (HIG11) |
| Add more clinic locations | - You can set up other places for the outreach clinic like Nairobi and other places for those travelling during that day [5.40] give many places where people can be checked for example the way I didn't make to attend the clinic in Meru I could have found some few hours to be checked somewhere else, [5.40]. (HIG1) |
| Mop- up clinics | - Schedule another clinic and I will come, inform me like one week before so that I will be prepared [3.01]. (HIG6) |
| Give each person a specific appointment slot | - Give people different appointment dates to avoid overcrowding (7:57) a particular number of appointment for a specific day of appointment (8:08) and come with a message to show u are supposed to be here today (8:17)dont give all people one day for appointment. (HIG3) |
| Schedule fewer people to attend each day | - N/A |
| **Awareness & Communications** | |
| SMS reminders, including on the day of the clinic | - You should also send text messages like the way you sent me a message last time [10.03] (HIG2) - Remind people about the clinic before the clinic day by sending text messages…Even though I was sick, if I could be reminded, I would try my best to come. (HIG5) - Send people text messages twice in the phone like one day before the clinic [10.38]. (HIG6) |
| Send a text to individuals who missed the outreach clinic to invite them to attend on another day | - N/A |
| Phone call reminder, especially for those who cannot read | - N/A |
| Use CHPs to remind people to attend | - The CHVs can be going home to home telling people ‘This is the day you should come to see the doctor' [4.26]. (HIG8) |
| Explain why attending clinic is important at the point of referral | - …also educate people about its importance as you convince them, also extra services like spectacles [6.09]. (HIG5) |
| Use media, posters, churches and radio broadcasts to remind people to attend | - Advertise using posters and media so that people can get the information, during the screening not everyone was aware and not all were screened, other people were not around so if you advertise everyone gets the information, [10.02]. (HIG1) - Advertise more like using stickers where there is movement of people like Makutano [9.35] some people didn't know about the screening and others also forgot [10.04] (HIG6) |
| Specify clinic dates and times | - N/A |
| Allow people to choose their appointment day | - N/A |
| Clarify what services are available at the outreach clinic and specify if there are any costs linked to these services | - N/A |
| **Norms, values & health beliefs** | |
| Separate younger people, women & children, and elderly people | - N/A |
| Other | - I can request for permission to go to the clinic for few minutes because people get permission even to go to hospital when they fall sick, [15.39]. (HIG2) |
